# Novel inhibitors that target bacterial virulence identified via HTS against intra-macrophage survival of *Shigella flexneri*

**DOI:** 10.1128/msphere.00154-23

**Published:** 2023-08-11

**Authors:** Marija Miljkovic, Sonia Lozano, Isabel Castellote, Cristina de Cózar, Ana I. Villegas-Moreno, Pablo Gamallo, Dolores Jimenez-Alfaro Martinez, Elena Fernández-Álvaro, Lluis Ballell, George A. Garcia

**Affiliations:** 1 Department of Medical Chemistry, College of Pharmacy, University of Michigan, Ann Arbor, Michigan, USA; 2 GSK Global Health Unit, Madrid, Spain; University of Rochester, Rochester, New York, USA

**Keywords:** *Shigella flexneri *2457T, shigellosis, high-throughput screening, THP-1 macrophages, intracellular, virulence, compound(s), hit(s)

## Abstract

*Shigella flexneri* is a facultative intracellular pathogen that causes shigellosis, a human diarrheal disease characterized by the destruction of the colonic epithelium. Novel antimicrobial compounds to treat infections are urgently needed due to the proliferation of bacterial antibiotic resistance and lack of new effective antimicrobials in the market. Our approach to find compounds that block the *Shigella* virulence pathway has three potential advantages: (i) resistance development should be minimized due to the lack of growth selection pressure, (ii) no resistance due to environmental antibiotic exposure should be developed since the virulence pathways are not activated outside of host infection, and (iii) the normal intestinal microbiota, which do not have the targeted virulence pathways, should be unharmed. We chose to utilize two phenotypic assays, inhibition of *Shigella* survival in macrophages and *Shigella* growth inhibition (minimum inhibitory concentration), to interrogate the 1.7 M compound screening collection subset of the GlaxoSmithKline drug discovery chemical library. A number of secondary assays on the hit compounds resulting from the primary screens were conducted, which, in combination with chemical, structural, and physical property analyses, narrowed the final hit list to 44 promising compounds for further drug discovery efforts.

The rapid development of antibiotic resistance is a critical problem that has the potential of returning the world to a “pre-antibiotic” type of environment, where millions of people will die from previously treatable infections. One relatively newer approach to minimize the selection pressures for the development of resistance is to target virulence pathways. This is anticipated to eliminate any resistance selection pressure in environmental exposure to virulence-targeted antibiotics and will have the added benefit of not affecting the non-virulent microbiome. This paper describes the development and application of a simple, reproducible, and sensitive assay to interrogate an extensive chemical library in high-throughput screening format for activity against the survival of *Shigella flexneri* 2457T-nl in THP-1 macrophages. The ability to screen very large numbers of compounds in a reasonable time frame (~1.7 M compounds in ~8 months) distinguishes this assay as a powerful tool in further exploring new compounds with intracellular effect on *S. flexneri* or other pathogens with similar pathways of pathogenesis. The assay utilizes a luciferase reporter which is extremely rapid, simple, relatively inexpensive, and sensitive and possesses a broad linear range. The assay also utilized THP-1 cells that resemble primary monocytes and macrophages in morphology and differentiation properties. THP-1 cells have advantages over human primary monocytes or macrophages because they are highly plastic and their homogeneous genetic background minimizes the degree of variability in the cell phenotype (1). The intracellular and virulence-targeted selectivity of our methodology, determined via secondary screening, is an enormous advantage. Our main interest focuses on hits that are targeting virulence, and the most promising compounds with adequate physicochemical and drug metabolism and pharmacokinetic (DMPK) properties will be progressed to a suitable *in vivo* shigellosis model to evaluate the therapeutic potential of this approach. Additionally, compounds that act via a host-directed mechanism could be a promising source for further research given that it would allow a whole new, specific, and controlled approach to the treatment of diseases caused by some pathogenic bacteria.

## INTRODUCTION

*Shigella flexneri* is a Gram-negative, facultative intracellular pathogen that causes shigellosis, a diarrheal disease in humans characterized by the destruction of the colonic epithelium. Shigellosis was the second leading cause of diarrheal mortality in 2016 across all age groups, increasing significantly among children younger than 5 years and elderly people (2). Due to *Shigella*’s fecal-oral route of transmission, the burden of shigellosis is predominantly felt in crowded communities in developing countries with insufficient access to adequate hygiene and sanitation as well as poorer overall health and nutritional status (2, 3). *Shigella* species are pathogens that are highly adapted to humans and some other primates with no known animal or environmental reservoir (4, 5).

The control of the burden of shigellosis is challenging for various reasons. First, it has been shown that a very low infective dose is needed to cause illness (6). Second, the variety of *Shigella* species and serotypes increases the possibility of reinfection because immunity to *Shigella* is serotype specific (7), which also makes it difficult to develop an effective vaccine. There is currently no approved vaccine for shigellosis (8, 9). Third, shigellosis can be treated with antibiotics that target Gram-negative pathogens; however, many strains of *Shigella* spp. are drug or multi-drug resistant, and this resistance has been rapidly growing over the past several decades (10, 11). Additionally, antibiotics are either bactericidal or bacteriostatic. One consequence of this is that it provides a selective pressure for the development of antibiotic resistance in the pathogen. Pathogen mutants which are resistant to the antibiotic will have a selective growth advantage over the drug-sensitive wild type. This resistance selection is not limited to antibiotic use to treat infections in humans. Environmental exposure of microbial pathogens to antibiotics due to their overuse/misuse in human medicine and their use in the food industry to promote growth and prevent disease has accelerated the development of resistance (12, 13).

Shigellosis is characterized by the ability of *Shigella* to invade, replicate, and spread within and between colonic mucosal epithelial cells (14). Briefly, *Shigella* disrupts the integrity of the epithelium allowing luminal bacteria to cross into the submucosa through microfold cells (M cells; specialized epithelial cells). After transcytosis, pathogens are released into an intra-epithelial pocket and engulfed by the residential macrophages (15). Inside the macrophages, *Shigella* ensures its survival by rapidly disrupting the phagocytic vacuole and inducing pyroptosis (16), which is accompanied by interleukin-1β (IL-1β) and IL-18 pro-inflammatory signaling (15, 17, 18). The pathogen, released from dying macrophages, subsequently invades the surrounding epithelium via the basolateral surface, again lyses the phagosome, and replicates intracellularly, before disseminating to adjacent cells, propelled by actin polymerization (15, 19). The *Shigella* invasion pathway involves the complex action of a large number of virulence factors encoded from the 210–220 kb virulence plasmid (20 – 23), along with some chromosomal genes (22). Sequence analysis of virulence plasmids from different *Shigella* strains has shown that the core of the plasmid is a 31-kb region, named “entry region,” which contains genes required for invasion of host epithelial cells and induction of macrophage pyroptosis and escape from macrophages (20, 21, 23). These genes encode components of a type III secretion system (T3SS), substrates of this apparatus (translocators and effectors), their chaperones, and two transcriptional activators (24). The primary regulator triggering the full expression of the *Shigella* invasion is the AraC-family transcription factor VirF (25, 26). Inactivation of VirF or other virulence factors eliminates, or significantly reduces, *Shigella* pathogenicity (27 – 29).

Our approach is a relatively new strategy (30, 31) to treat bacterial infections via inhibiting virulence factors that are required for infection rather than an antibacterial approach. This approach has three potential advantages: (i) resistance development should be minimized due to the lack of growth selection pressure when targeting virulence rather than growth, (ii) no resistance due to environmental antibiotic exposure should be developed since the virulence pathways are not activated outside of host infection, and (iii) the normal intestinal microbiota, which do not have the targeted virulence pathways (32), should be unharmed. This latter point is important as administration of broad-spectrum antibacterials often eliminates the normal intestinal microbiota, leading to dangerous opportunistic infections, e.g., *Clostridium difficile* colitis (33). Our objective was to identify compounds that block the *Shigella* virulence pathway, preventing *Shigella* from triggering pyroptosis and escaping from macrophages, which will then eradicate the bacteria. To achieve this objective, we have developed novel, extremely high-throughput screening (HTS) methodology to identify compounds active against intracellular survival of *Shigella* in THP-1 macrophages and completed an HTS campaign of the GlaxoSmithKline (GSK) 1.7 M compound screening collection. Secondary screening of *Shigella* antibacterial activity and inhibition of *Shigella* propagation in Caco2 cells, to focus on those hit compounds that are “antivirulence” rather than antibacterial, combined with chemical property analyses yielded 44 very promising compounds.

## RESULTS

### *Shigella* intracellular survival in THP-1 macrophage assay (intra-macrophage survival assay) and high-throughput screen development

We took advantage of *Shigella*ʼs ability to ensure its survival in macrophages by rapidly inducing pyroptosis, which involves the expression of several virulence factors, to develop an HTS method for the discovery of compounds having an adverse effect on virulence. Consequently, *S. flexneri* cannot trigger pyroptosis and escape from macrophages, which will then eradicate the bacteria. A novel protocol was developed to identify compounds active against intracellular bacteria by using THP-1 human monocytes infected with *S. flexneri* 2457T-nl strain, expressing a luciferase reporter gene for primary identification. In addition, the protocol efficiency was enhanced by throughput optimization to the 1,536-well plate format (1,408—maximum number of test compounds per plate), which allows for the screening of ~56 K compounds in one round of assay (~40 plates). In the protocol, THP-1 differentiation, infection, gentamicin treatment, and incubation of the infected cells with the compounds to be tested were performed on the same day. After 18 hours of incubation, the luciferase-based readout was performed. A schematic representation of the optimized HTS methodology, time required to perform one round of assay, and method efficiency is shown in Fig. S1.

Large-scale production of THP-1 cells was achieved by using roller bottles with up to 0.5 L of total volume each, which is significantly different from microplate intracellular assays. Maintaining cells in these conditions allows uniform growth, differentiation, and infection of THP-1 macrophages in a single step and represents a key strategy to improve assay robustness by decreasing well-to-well variation. The differentiation of THP-1 monocytes into macrophages was conducted with phorbol 12-myristate 13-acetate (PMA) at a final concentration of 40 ng/mL (34, 35). PMA treatment of THP-1 cells leads to a more mature phenotype with a lower rate of proliferation, higher levels of adherence, higher rate of phagocytosis, and increased cell-surface expression of CD11b and CD14 (36).

Experiments performed for the purpose of HTS optimization are shown in Fig. S2. Intracellular bacterial loads were quantified by the traditional colony-forming unit (CFU) method (Fig. S2A and B). A range of multiplicities of infection (MOIs bacteria:cells—50:1, 20:1, 8:1, and 1:1) and infection times (0.5–24 hours) were tested for *S. flexneri* 2457T-nl. The conditions that worked best were infections for 2 hours at an optimum MOI of 8:1. Although centrifugation can remove a high percentage of extracellular bacteria, the remnant is still significant enough to confound the assay. Therefore, we used gentamicin treatment after invasion to eliminate any extracellular bacteria. The importance of gentamicin treatment in this protocol is shown in Fig. S2C.

**Fig 1:**
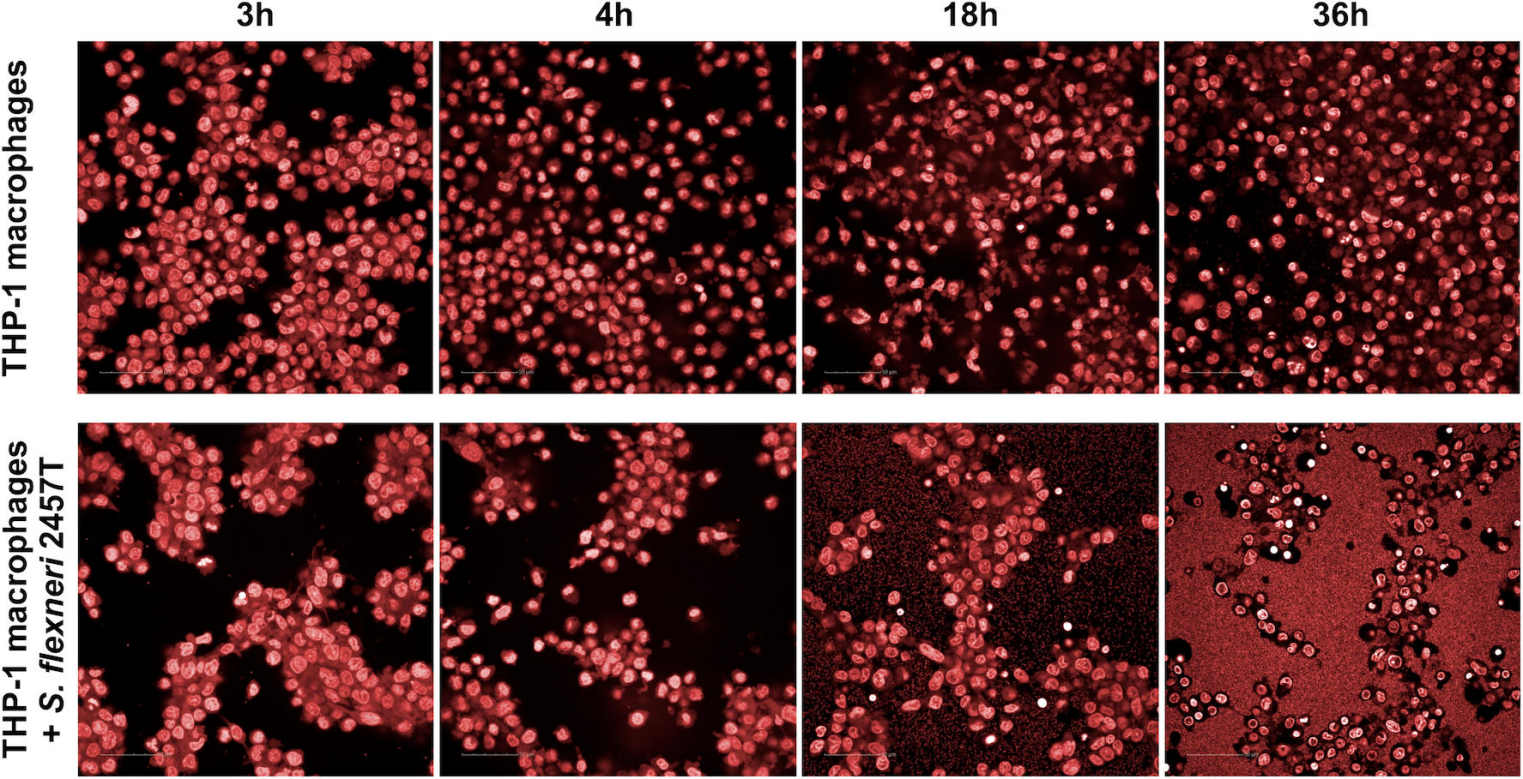
THP-1 macrophages ± treatment with *S. flexneri* at 3, 4, 18, and 36 hours after invasion. Human monocyte THP-1 PMA-differentiated macrophages were stained with 5 µM DRAQ5 far-red fluorescent (Biostatus, Shepshed, Leicestershire, United Kingdom) with 647 nm excitation wavelengths in dark. Single plane images were acquired on an Opera Phenix high-content screening system (PerkinElmer, Waltham, MA, USA) using a 20× water immersion objective in confocal mode. Three fields per well were acquired for reliable statistical analysis, and images were analyzed using Harmony (PerkinElmer, Waltham, MA, USA) HC imaging software.

It was crucial to define assay conditions where, with no added compound, the *S. flexneri* do trigger pyroptosis in and escape from the macrophages. To determine the optimal incubation time of the infected macrophages with the compounds to be tested, we measured Caspase-1 activity at different time points (Fig. S2D), which indicated the level of macrophage pyroptosis under conditions in which compounds should exert their effects. The results show that after 16 hours of incubation, Caspase-1 activity has plateaued for the infected macrophage sample and is declining for the non-infected THP-1 sample (control). Since there is no significant difference in Caspase-1 activity at times greater than 16 hours in the infected macrophages sample, we selected an incubation time of 18 hours for the HTS protocol. The selection of 18 hours was also supported by observations of infected and non-infected macrophages on the confocal microscope (Fig. 1). Samples were taken at different time points after invasion, and THP-1 macrophages were stained using DRAQ5 far-red fluorescent DNA dye. The results show significantly different distribution between infected THP-1 macrophages and macrophages without bacteria (control). It is noticeable that infected macrophages form cell aggregates, presumably as a protection against bacteria.

### *Shigella* intra-macrophage survival high-throughput screen validation

Evaluation of *Shigella* intra-macrophage survival assay robustness was assessed by calculating the Z′ values from 10 plates (1,536-well plate format) in independent runs of assay using only dimethyl sulfoxide (DMSO, 0.5% vol/vol) as a negative control (no effect on *Shigella* growth) and moxifloxacin (20 µM) as a macrophage permeable positive control (at 20 µM moxifloxacin, there was no detectable bacterial survival). Moxifloxacin and DMSO were used as controls on each plate in the HTS to monitor assay quality through determination of Z′ as well as for normalizing the data on a per-plate basis (see Data analysis in Materials and Methods). Across these 10 plates, a Z′ factor value of 0.58 (±0.1) was observed, which is well above the acceptable threshold (>0.4) for conducting an HTS (Fig. S3). In the next step of method validation, *in vitro* activities of selected commercially available antibiotics against Gram-negative bacteria were tested. The IC_50_ (luciferase assay) values for ciprofloxacin, mecillinam, pivmecillinam, azithromycin, ceftriaxone, and PF-5081090 for *S. flexneri* 2457T in survival in THP-1 macrophage assays are shown in Table S1 along with their minimum inhibitory concentrations (MICs, resazurin assay). Among the panel of tested antibiotics, the MIC of pivmecillinam was very similar to its survival IC_50_, while the MICs for ciprofloxacin, mecillinam, ceftriaxone, and PF-5081090 are 2–5 times higher, azithromycin 12 times higher than their MICs against *S. flexneri* 2457T (Table S1). *S. flexneri* 2457T-nl intra-macrophage survival methodology was further validated using the set of ~10 K compounds, and the assay parameters were calculated to monitor the quality of the resulting data. The Z′ factor for this pilot screen was 0.77 (±0.02).

### The HTS progression cascade

**Fig 2:**
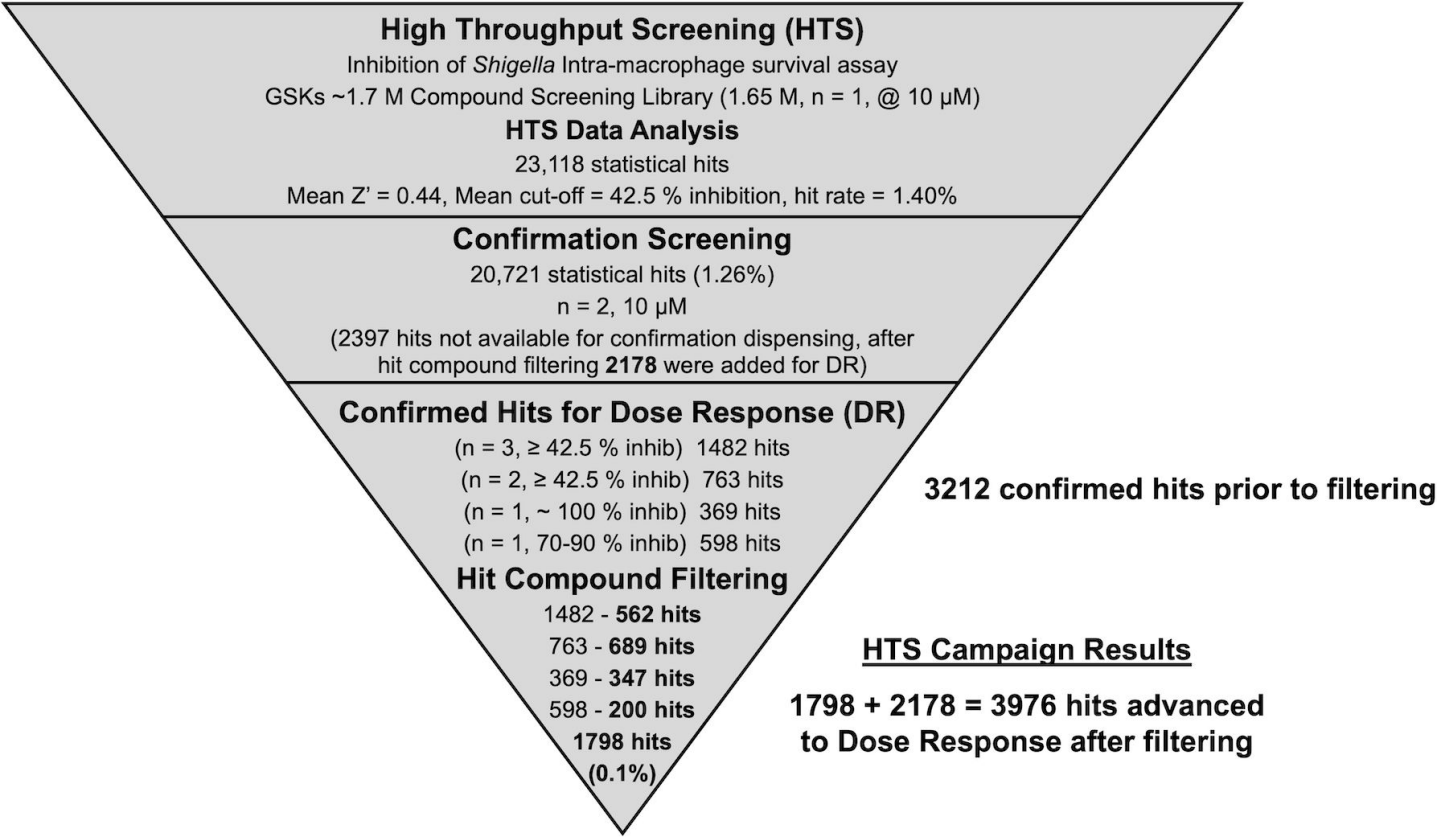
The HTS progression cascade. Diagram of the primary screening, confirmation, and hit compound filtering process with aggregate data on hits at each stage.

**Fig 3:**
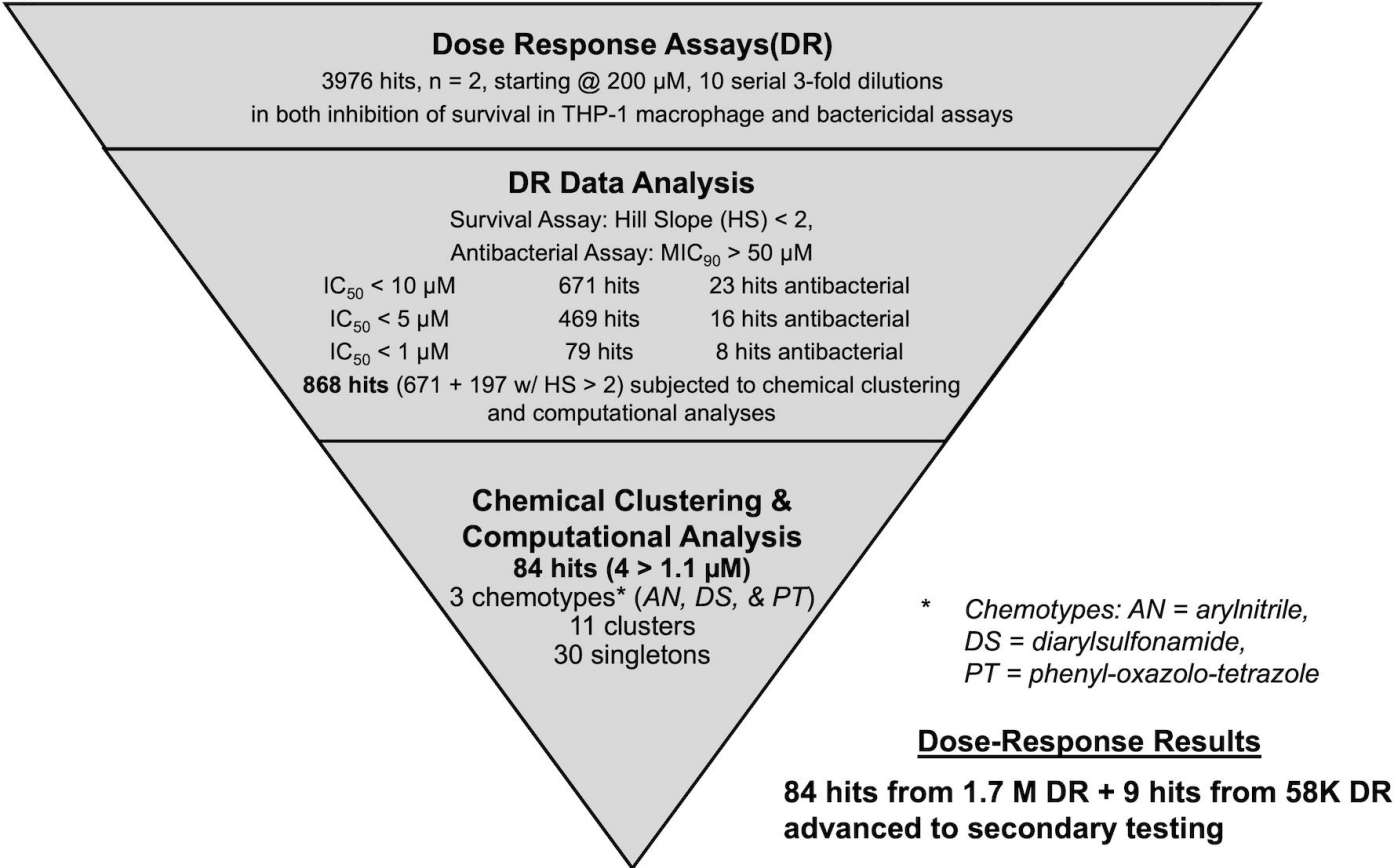
Dose-response progression cascade. Diagram of the dose-response screening in both *Shigella* intra-macrophage survival and *Shigella* antibacterial assays and chemical filtering with aggregate data on hits at each stage. *n* = the number of replicates.

A summary of the HTS progression cascade is shown in Fig. 2 and 3 followed by secondary assay screening shown in Fig. 5. HTS workflow to identify compounds with activity against *Shigella* intra-macrophage survival contains the following steps: (i) primary and confirmation screens and hit compound filtering shown in Fig. 2, (ii) survival and antibacterial dose-response assays, hit selection based on antibacterial effect and chemical clustering including computational analysis shown in Fig. 3, and (iii) hit selection based on inhibition of *Shigella* propagation in Caco2 cells, lack of Nano-Luciferase (Nano-Luc) inhibition (no interference with the assay readout), and lack of cytotoxicity on THP-1 macrophages, Caco2, and HepG2 cells (Fig. 5).

**Fig 4:**
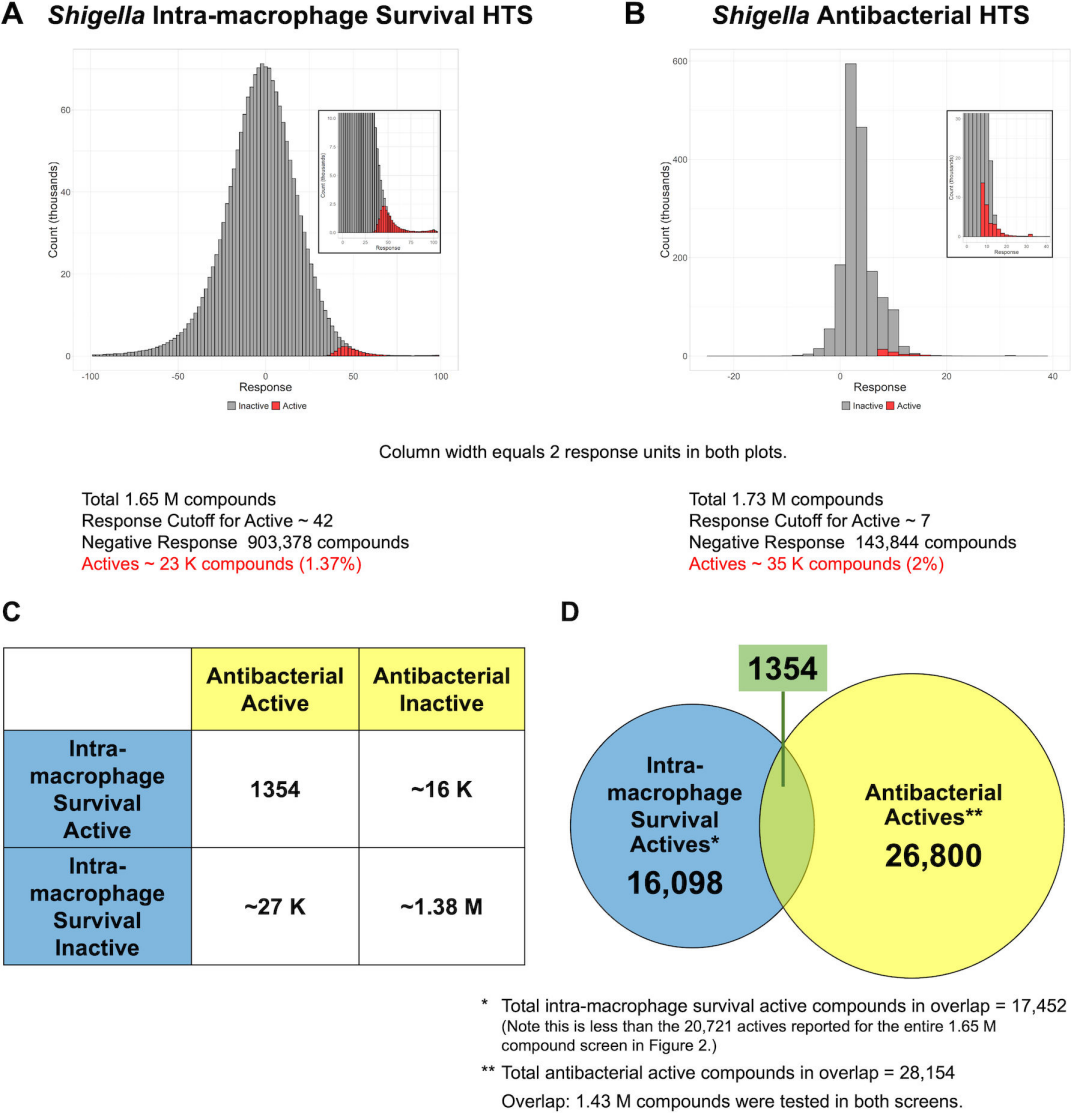
Comparative analyses of HTS results for *Shigella* intra-macrophage survival and *Shigella* antibacterial screens. (A) Distribution of the percent inhibition in the HTS of *Shigella* intra-macrophage survival activity. (**B**) Distribution of the percent inhibition in the HTS of *Shigella* antibacterial activity. Compounds assigned to be inactive and active are marked gray and red, respectively. The numbers of active compounds (in red) are averages over entire HTS campaigns. The plots were created using “ggplot2” (https://ggplot2.tidyverse.org/; which is based on statistical programming language R). (**C**) Numbers of active and inactive compounds in each HTS. (**D**) Venn diagram of active compound overlap. Numbers in C and D are out of the ~1.43 M compounds tested in both HTS (overlap).

**Fig 5:**
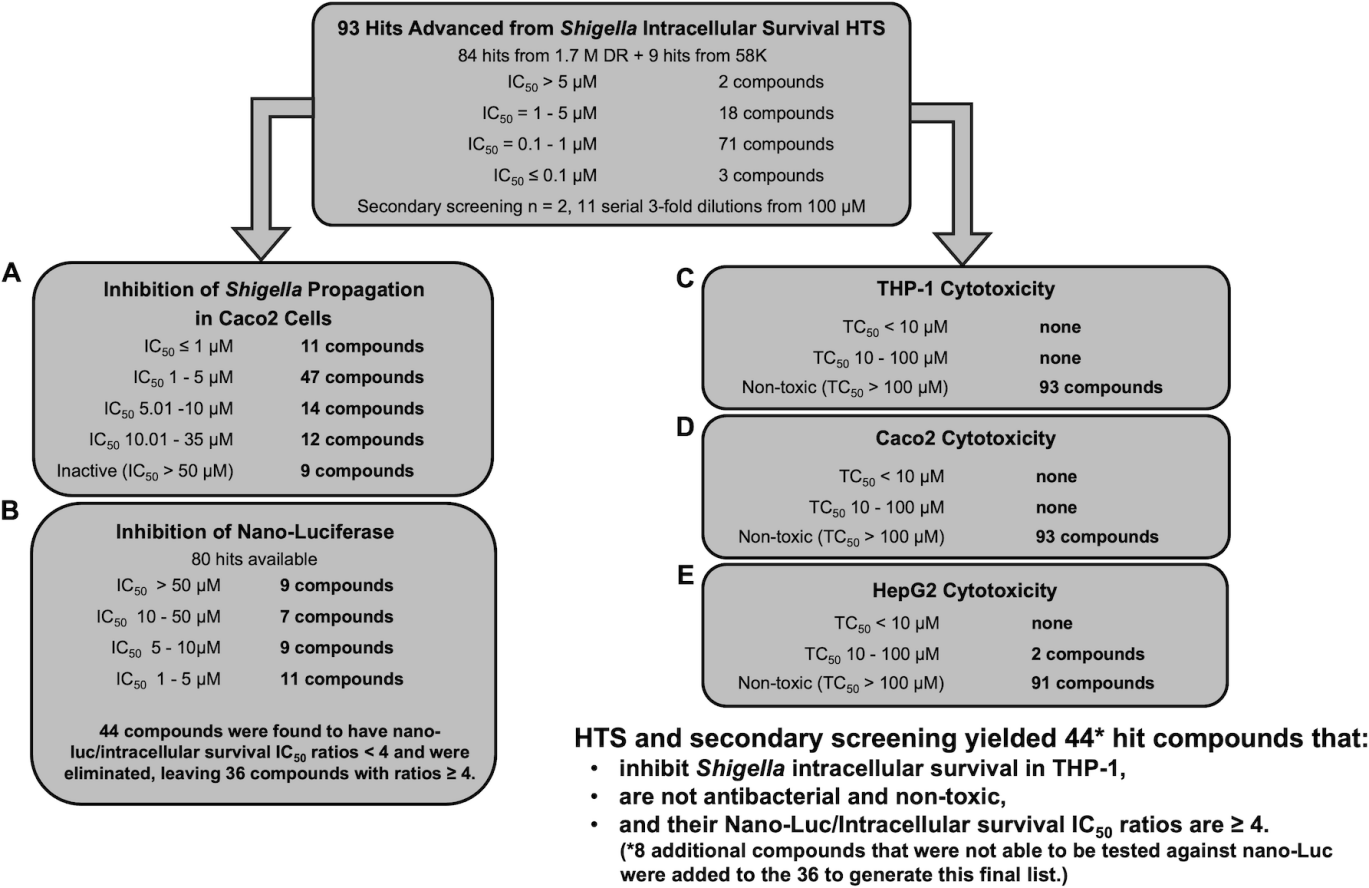
Results of secondary assay screening. (A) The inhibition of *Shigella* propagation in Caco2 cells assay, (**B**) Nano-Luc inhibition, **(C**) THP-1 cytotoxicity, (**D**) Caco2 cytotoxicity, and (E) HepG2 cytotoxicity.

#### Primary screening

To identify novel and potent chemical matters that block *Shigella* virulence, the 1.7 M GSK screening collection (actually 1.65 M, at 10 µM) was screened in the *Shigella* intra-macrophage survival high-throughput assay. The results of this HTS gave 23,118 initial (statistical) hits with an overall hit rate of 1.4%. Compound distribution by % inhibition of *Shigella* intra-macrophage survival in the HTS is shown in Fig. 4A. The average Z′ value for the 53 rounds of this HTS was 0.44, and the inhibition cut-off was 42.5%. For comparison, in Ballell et al. (37), 1.9 M compounds were screened in an antimycobacterial phenotypic screening campaign against *Mycobacterium bovis* BCG. Using a cut-off of 45.2% inhibition yielded 62,000 primary hits (hit rate of 3.3%). In a more recent study (38), out of 40,560 compounds tested, 731 compounds exhibited ≥50% decrease in the fluorescence of mycobacteria-infected THP-1 cells (hit rate of 1.8%).

#### Confirmation screening

To exclude false positive hits and/or hits with THP-1 macrophage toxicity, 20,721 hits from the primary screen (2,397 of the primary screen hits were not available for confirmation dispensation) were subjected to confirmation screening in duplicate at 10 µM (Fig. 2). The confirmation screening of these hits yielded a subset of 1,482 confirmed hits that were active in all three assays (primary screen and both confirmation assays) and 763 confirmed hits that were active in two of the three repetitions. These results indicate that re-confirmation was obtained in ~10% of the primary screen hits. To this set of re-confirmed hits were added 369 and 598 statistical hits that were considered active only based on the primary screen with inhibition of 90%–110% and 70%–90%, respectively. A total of 3,212 hits passed the confirmation screening.

#### Hit compound filtering

The 2,397 hits (from the primary screen hits) that had to be excluded from the confirmation screening were added to the 3,212 confirmed hits and subjected to the hit compound filtering process, resulting in 3,976 hits with promising properties and structural novelty that were advanced to dose-response testing (Fig. 2).

#### *Shigella* intra-macrophage survival dose-response assay

The 3,976 hits were further characterized by dose-response in the *Shigella* intra-macrophage survival assay performed in duplicate (the average Z′ value across both sets was 0.59 ± 0.02). Analysis of the results yielded 868 hit compounds with IC_50_ <10 µM; of those, 671 hit compounds had Hill slopes <2.0 [in HTS, Hill slopes larger than 2 are often indicative of false positives due to compound aggregation (39), hence our cutoff of Hill slope <2.0]. More detailed results of the intracellular dose-response studies are presented in Table 1.

**TABLE 1:** The overall results of *Shigella* intra-macrophage survival and *Shigella* antibacterial dose-response studies

Selection criteria^*a*^	Numbers of compounds	
	*Shigella* intra-macrophage survival	*Shigella* antibacterial^*b*^
IC_50_ <50 µM	1,272	95
IC_50_ <10 µM	868^*c*^	86
IC_50_ <5 µM	636	76
IC_50_ <1 µM	129	36
IC_50_ <50 µM and slope <2	988	29
IC_50_ <10 µM and slope <2	671	23
IC_50_ <5 µM and slope <2	469	16
IC_50_ <1 µM and slope <2	79	8

^*a*^ IC_50_ in *Shigella* intra-macrophage survival assay, slope = Hill slope of dose-response curve fit.

^*b*^ Number of compounds that gave a determinable MIC.

^*c*^ Number of compounds advanced to chemical clustering and computational analyses.

#### Comparative analysis of *Shigella* intra-macrophage survival and *Shigella* antibacterial HTS results

We performed a comparative analysis of the results for the *Shigella* intra-macrophage survival HTS and the *Shigella* antibacterial HTS. The antibacterial HTS had an average Z′ value of 0.75, and the inhibition cut-off was 25.5% (40). Figure 4A and B show the response distributions (i.e., the numbers of compounds within two response unit bins) for each HTS. The response distribution for the antibacterial HTS was much narrower than that for the intra-macrophage survival HTS. Interestingly, the response distribution for the intra-macrophage survival HTS centered close to zero response, with a negative response for ~900 K compounds. The response distribution for the antibacterial HTS had only ~144 K compounds with a negative response. Figure 4C and D show the numbers of active and inactive compounds in each HTS and the intersection of the two HTS (e.g., compounds active in both HTS) for the 1.43 M compounds that were tested in both screens. Fig. S4 details the numbers of compounds tested in each of the screens.

#### Hit reduction based on antibacterial activity

Of the 3,976 hits that were advanced to dose-response testing, 98% were determined to be inactive in the *Shigella* antibacterial HTS project performed earlier at GSK (40), and consequently, dose-response studies were not performed for those compounds in that study. However, the thresholds for activity determination in the intra-macrophage survival (45.5%) and antibacterial assays (25.5%) were different as noted above. To accurately compare the intra-macrophage survival and antibacterial activities for 3,976 hits, dose-response studies to determine the MICs for these hits were conducted (Table 1). Analysis of the results indicated that only 157 of the 3,976 hits had MICs <50 µM. Within the prioritized 671 hits from the intra-macrophage survival dose-response study, 23 hits have antibacterial activity against *S. flexneri* 2457T, leading to 648 likely virulence-selective hits.

#### Chemical clustering and computational analysis

All hits with IC_50_ <10 µΜ (*n* = 868) in the *Shigella* intra-macrophage survival dose-response assay were clustered by chemical structure. After analysis, 84 compounds (belonging to 11 structural clusters and 30 singletons) were selected (Fig. 3). Half of the selected compounds belong to two major chemotypes (see Structural analysis below). In addition, nine hits from the 58 K pilot screen (three belonging to one cluster and six singletons) were added to previous hits, giving 93 hits to advance to secondary screening.

### Secondary assay screening

Secondary assays including Nano-Luc inhibition, inhibition of *Shigella* propagation in Caco2 cells, and cytotoxicity on THP-1, Caco2, and HepG2 cells were conducted on the 93 advanced hits to more precisely determine the possibility of targeting virulence factors and identify undesirable side effects, respectively (Fig. 5).

#### Elimination of likely false positives due to Nano-Luc inhibition

Of the 93 hits, 80 were available for screening for inhibition of the Nano-Luc reporter to eliminate false positives (Fig. 5A). Of the hits that were advanced after dose-response testing, 44 were found to have Nano-Luc/intracellular survival IC_50_ ratios <4 and were eliminated. Eight of the 93 hits that were not available for Nano-Luc screening were combined with the 36 remaining hits for a total of 44 final hits.

#### Hit reduction based on inhibition of *Shigella* propagation in Caco2 cells

Analysis of the *Shigella* propagation in Caco2 cells assay results showed that 9 of the 93 hits advanced to secondary assay screening were inactive, leaving 84 hits active in both the *Shigella* intra-macrophage survival and *Shigella* propagation in Caco2 cell assays (Fig. 5B).

#### Hit reduction based on cytotoxicity

In parallel, we conducted cytotoxicity testing in duplicate on the entire set of 93 hits that were advanced to secondary assay screening. In the first step, we determined compound cytotoxicity against the human macrophage THP-1 host cells. All 93 compounds had TC_50_ >100 µM and were classified as non-toxic (Fig. 5C). In the second step, we probed hit compound cytotoxicity on Caco2 cells, where again no toxic compounds (TC_50_ >100 µM) were observed (Fig. 5D). In the last step, cytotoxicity on HepG2 cells was tested (Fig. 5E). Results show weak cytotoxicity of two compounds (TC_50_ ~85 µΜ), while 91 compounds had TC_50_ >100 µM. All 44 compounds were retained.

### Priority scoring results

The scoring system is a weighted combination of scores for activity, physical parameters, and “off-target” effects. Each of these sub-scores is weighted (by a “metric normalization factor” (NF) and a “priority weighting factor” (WF)) combinations of individual property values and shown in Table 2. The priority scores for the hit compounds are presented in Table 3. Only the final 44 hits were scored. Certain scoring rules were introduced: (i) since the values for fasting state simulated intestinal fluid (FaSSIF) do not correlate with the water solubility results, weighting value of this criterion was zero; FaSSIF can be considered independently later in the progression process, and (ii) the ranking weighting values of the cytotoxicity were zero, given that none of the 44 hits exert cytotoxic effects (TC_50_ <10 µΜ) on any tested cell line.

**TABLE 2:** Ranking scoring system and parameters used to prioritize compounds

Overall score = A/AS + B × PPS – C × OTS					
	Parameters^*a*^		WV^*b*^	NF	WF
**Activity score (AS)**	AS	A	10	5	2
1/(A1 × THP-1 IC_50_)	*THP-1 IC_50_*	*A1*	1	1	1
**Physical parameter score (PPS)**	PPS	B	5	1	5
(−B1 × MW) ± (B2 × PFI)^*c*^ + (B4 × LE) + (B5 × LLE) + (B6 × FaSSIF)	*MW*	*B1*	0.001	0.001	1
	*PFI*	*B2*	1	1	1
	*LE*	*B3*	1	1	1
	*LLE*	*B4*	1	1	1
	*FaSSIF*	*B5*	0	1	0
**“Off-target” score (OTS)**	OTS	C	2	1	2
(C1 × THP-1 TC_50_) + (C2 × Caco2 TC_50_) + (C3 × HepG2 TC_50_) + (C4 × hERG) − (C5 × IFI)	*THP-1 Tox*	*C1*	0	1	0
	*Caco2 Tox*	*C2*	0	1	0
	*HepG2 Tox*	*C3*	0	1	0
	*hERG*	*C4*	0.5	1	0.5
	*IFI*	*C5*	1	1	1

^*a*^ Parameters are as described in Materials and Methods. Manually removed compounds that have Nano-Luc/Intracellular survival IC_50_ ratios <4 (*n* = 53). IC_50_ values are in µM.

^*b*^ Weighting value, WV = NF × WF.

^*c*^ +B2 if PFI (4–7), –B2 if PFI (<4 or >7).

**TABLE 3:** Final 44 hit compounds ranked by prioritization scores

Compound ID	Priority scoring				Survival inhibition	Propagation in Caco2	Nano-Luc interference
	Activity score	Physical parameter score	“Off-target” score	Overall score	IC_50_ (µM)	IC_50_ (µM)	IC_50_ (µM)
**AN1-152-02**	**254.1**	**−4.4**	**−2.0**	**2,515**	**0.0039**	**0.3**	**3.7**
**AN-5-02**	**11.2**	**11.7**	**−8.3**	**154**	**0.09**	**2.1**	**0.78**
**AN1-152-01**	**3.2**	**12.3**	**−1.5**	**91**	**0.31**	**2.7**	**25**
S-86-01	3.7	9.8	−4.0	78	0.27	1.0	1.7
**AN1-152-03**	**2.1**	**11.7**	**−0.6**	**78**	**0.48**	**1.7**	**62**
**AN1-152-05**	**10.4**	**−6.0**	**−0.6**	**73**	**0.10**	**0.3**	**12**
S-281-01	1.6	10.9	−0.5	69	0.64	24	28
DS-64-06	2.3	9.1	−2.0	64	0.44	3.1	9.5
**AN2-45-01**	**0.9**	**10.4**	**−0.7**	**60**	**1.1**	**15**	^*a*^
**AN2-45-02**	**1.1**	**9.5**	**−1.4**	**56**	**0.89**	**14**	^*a*^
DS2-187-02	0.8	10.0	−1.3	56	1.2	4.4	7.4
DS2-147-01	1.0	9.1	−0.2	55	1.0	6.4	5.6
S-378-01	1.0	9.2	−3.1	50	1.0	6.6	^*a*^
S-53-01	5.8	−6.4	−1.7	23	0.17	1.5	1.2
AN1-152-07	4.7	−5.4	−0.9	18	0.21	0.2	27
58K-S-05	0.7	1.9	0.0	16	1.5	9.5	7.4
58K-S-04	0.6	2.7	−1.5	16	1.8	2.3	^*a*^
58K-S-01	0.5	2.0	0.0	15	2.1	9.0	^*a*^
S-136-01	1.6	1.6	−6.8	11	0.62	3.1	3.0
S-169-01	1.7	−2.8	−1.5	0	0.59	21	12
PT-297-02	3.7	−6.4	−3.3	-2	0.27	1.2	4.9
S-87-01	1.3	−3.3	−2.0	-8	0.78	8.8	9.8
S-82-01	1.0	−2.0	−4.1	-8	1.0	19	19
AN1-152-08	2.3	−6.3	−0.2	-9	0.43	1.9	^*a*^
DS2-187-03	1.4	−4.4	−1.5	−11	0.70	3.0	^*a*^
PT-297–03	2.0	−5.8	−1.4	−12	0.50	3.0	8.1
AN1-152-06	2.1	−6.4	−0.5	−13	0.49	3.9	100
PT-297-01	2.3	−6.4	−3.5	−16	0.43	2.1	8.3
S-10-01	2.4	−7.9	−0.6	−17	0.41	0.5	11
S-185-01	1.4	−5.3	−2.5	−17	0.70	4.3	3.8
DS-61-01	2.0	−6.3	−3.5	−19	0.50	2.3	2.2
AN1-152-09	1.4	−6.4	−0.2	−19	0.73	10	^*a*^
DS-299-02	1.3	−3.8	−6.5	−20	0.80	2.5	4.6
S-28-01	1.0	−4.6	−3.7	−20	0.99	4.5	5.0
AN1-152-04	1.8	−7.3	−1.1	−21	0.57	6.2	100
S-10-03	1.6	−7.7	−2.4	−27	0.62	1.1	^*a*^
S-134-02	1.5	−7.1	−3.9	−29	0.68	4.9	5.1
S-316-01	0.8	−7.0	−0.9	−29	1.3	1.9	100
DS-90-01	0.8	−7.1	−2.7	−33	1.2	10	65
DS1-32-04	2.5	−8.4	−9.9	−37	0.41	3.8	3.1
AN1-77-01	1.3	−7.8	−6.0	−38	0.79	14	100
DS1-32-05	1.8	−8.8	−7.8	−41	0.56	3.1	3.0
AN-30-01	N/A				0.88	Inactive	100
AN1-152-10	N/A				0.24	Inactive	50
			**Mean**	73	0.69	5.8	26
			**SD**	389	0.44	5.9	34
			**Max^*b*^**	2,515	2.1	24	100
			**Min**	−41	0.0039	0.24	0.78

^*a*^ Unable to be tested for Nano-Luc interference.

^*b*^ Max not included in mean and standard deviation (SD).

^*c*^ AN chemotypes in top ten ranked compounds are in bold.

### Structural analyses

**Fig 6:**
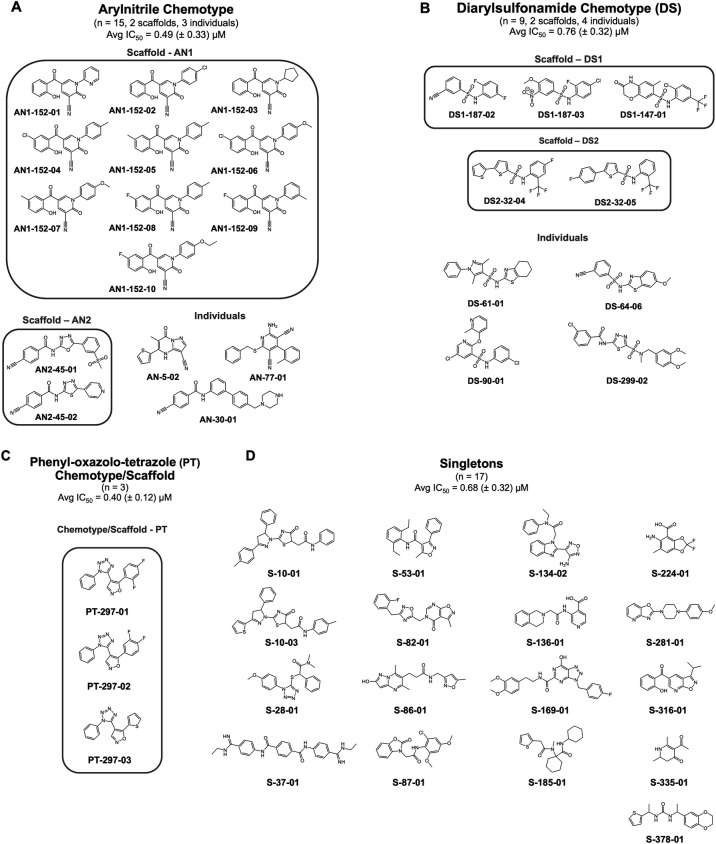
One minor and two major chemotypes identified by structural clustering and chemical property analyses. (**A**) AN chemotype, (**B**) DS chemotype, (**C**) PT chemotype, and (**D**) singletons.

Among the 44 scored hit compounds, three major chemotypes, arylnitriles (AN), diarylsulfonamides (DS), and phenyl-oxazolo-tetrazoles (PT) accounting for 15, 9, and 3 compounds, respectively, were identified via a manual review of the chemical clustering analysis. The three chemotypes contain two AN, two DS, and one PT-specific chemical scaffolds and three AN and four DS individual analogs (compounds that fit into the chemotype but not into one of the identified scaffolds). The 17 remaining scored hit compounds represent discrete chemotypes (e.g., singletons). The three chemotypes and scaffolds along with the aggregate primary screen IC_50_s for each scaffold are shown in Fig. 6. Seven of the top 10 scored compounds are in the AN chemotype, four AN1, two AN2, and one AN singleton (Table 3, highlighted in bold).

## DISCUSSION

Our goal was to develop and utilize an assay to find compounds that block the *Shigella* virulence pathway to minimize resistance development (due to lack of growth advantage selection) and avoid harming non-pathogenic enteric microbiota. In the presence of a virulence inhibitor, the *Shigella* should not be able to trigger pyroptosis and escape from macrophages, which will then eradicate the bacteria. We chose to utilize a combination of phenotypic assays to interrogate the 1.7 M compound screening collection subset of the GSK drug discovery chemical library. Building upon previous reports of a THP-1 human monocyte-based assay to identify compounds active against intracellular *Mycobacterium tuberculosis* (35) and *Mycobacterium bovis* BCG (37) and a RAW264.7 macrophage-based screen to identify antibacterial compounds selective for intracellular *Salmonella typhimurium* (41), we developed our primary assay involving screening compounds for the ability to inhibit the intracellular survival of *S. flexneri* 2457T-nl in THP-1 macrophages. It was crucial to define assay conditions where, with no added compound, *Shigella* triggers pyroptosis in and escapes from the macrophages (as previously reported [42]). As shown in Fig. 1, untreated *Shigella* do indeed escape into the surrounding media where they replicate dramatically. After pilot screening validation of our primary HTS assay, we conducted an HTS and confirmation screening of 1.65 M compounds (Fig. 2). Dose-response testing in this primary assay was conducted on 3,976 confirmed hits.

Prior to our primary screen, an antibacterial HTS of the same 1.7 M compound library against *S. flexneri* 2457T had been conducted by researchers at GSK (40). We performed a comparative analysis of these two very large screens. It is interesting that ~55% of the 1.7 M compounds gave a negative response in our primary *Shigella* intra-macrophage survival screen, while less than 10% of the compounds gave a negative response in the antibacterial screen (Fig. 4A and B) (note that a negative response indicates that the *Shigella* are growing *faster* than the control). The reason for the negative response observed in the *Shigella* intra-macrophage survival screen is not clear. It does seem likely that compounds that impair macrophage function may facilitate *Shigella* escape into the media. In the supplemented RPMI 1640 medium, *Shigella* can rapidly replicate to higher densities than in the absence of compounds that impair macrophage function, resulting in the negative response. This large negative response was not observed in the *M. bovis* BCG HTS (37) (personal communication from one of the authors). It should be noted that mycobacteria cannot replicate in the media used for the intra-macrophage survival screens; therefore, compound impairment of the macrophages could not result in enhanced extracellular growth of the mycobacteria. Additionally, the presence of macrophage aggregates in some wells can lead to a higher starting number of intracellular *Shigella*, leading to a negative response. Another potential factor is that although the screens use the same parent strain (*S. flexneri* 2457T), the intra-macrophage survival screen strain contains the Nano-Luc plasmid. Additionally, the two screens have different readouts: resazurin for the antibacterial HTS and luminescence for intra-macrophage survival HTS. The actual overlap of these two large screens was 1.43 M compounds (Fig. 4C and D), and surprisingly, only 1,354 compounds were active in both screens. This suggests that, like gentamicin, the vast majority of the antibacterial hit compounds cannot access the *Shigella* inside the macrophages.

The three most likely mechanisms for inhibition of *Shigella* intra-macrophage survival are antibacterial activity, inhibiting *Shigella* virulence, and protecting the macrophages from pyroptosis or activating the macrophages to eliminate the *Shigella*. Using an antibacterial dose-response assay, we identified only 23 of the 3,976 compounds with MICs <50 µM. This is consistent with the HTS comparative analysis described above. After chemical/structural clustering and physical property analyses, yielding 84 hit compounds (Fig. 3), we performed another intracellular screen monitoring the inhibition of *Shigella* propagation in Caco2 cells. Only nine compounds were inactive in the propagation in Caco2 screen. It is possible that these compounds act via a host-directed mechanism to activate and/or protect the macrophages. This will require further study.

To factor out compounds that may be false positives due to Nano-Luc inhibition, the 93 hits advanced from the intracellular survival screen were tested in a Nano-Luc assay as described in Materials and Methods using the same *S. flexneri* 2457T-nl reporter strain as in the initial HTS (Fig. 6). Thirty-six compounds were found to have Nano-Luc/intracellular survival IC_50_ ratios ≥4 and were advanced to further studies. Additionally, eight compounds that were not available for nano-luciferase testing were added to yield a set of 44 validated hit compounds. Among the compounds eliminated due to Nano-Luc inhibition, there were 18 members of the DS chemotype. It should be noted that while these compounds do interfere with the Nano-Luc reporter, they could also be inhibitors of *Shigella* intracellular survival in macrophages. Further studies with an assay that does not involve a luciferase readout are necessary.

To ensure that the compounds were suitable for advanced studies, cytotoxicity assays were performed on THP-1 macrophages, Caco2, and HepG2 cells with the hit compounds resulting from the survival and antibacterial screens. Reassuringly, only two compounds had moderate HepG2 toxicity (10–100 µM), and none had THP-1 or Caco2 toxicity. We developed a scoring system that is independent of chemical structure to rank the entire set of 44 confirmed hit compounds (Table 2). The overall scores and sub-scores are shown in Table 3. It is not unexpected that the off-target sub-score has a relatively small contribution to the overall score, given that toxic compounds were screened out of the final list. The physical parameter scores vary from approximately −10 to 10, largely due to predicted solubility.

Chemical clustering and manual analysis of the compound structures revealed that 24 of the 44 confirmed hit compounds belong to two chemotypes, AN and DS, each of which contains clusters of more closely related compounds sharing a specific chemical scaffold (Fig. 6A and B). A third minor chemotype of a single scaffold, PT, consisting of three compounds was also identified [Fig. 6C along with 17 individual “singleton” compounds (Fig. 6D)].

**Fig 7:**
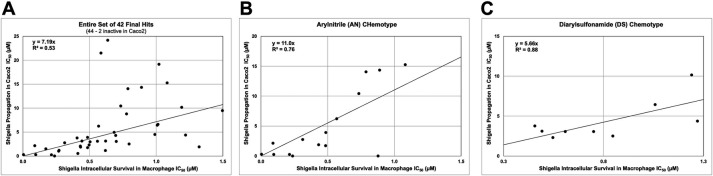
Correlations of IC_50_s for *Shigella* intra-macrophage survival and inhibition of *Shigella* propagation in Caco2 cell assays. (**A**) Entire set of 42 final hits (44, 2 inactive in Caco2), (**B**) AN chemotype (15 compounds), (**C**) DS chemotype (9 compounds).

Across the set of 44 compounds, the IC_50_s for *Shigella* intra-macrophage survival and *Shigella* propagation in Caco2 correlated weakly (*R* ^2^ = 0.53, Fig. 7A). This weak correlation could be due in part to the relatively small variance in the intra-macrophage survival IC_50_s making it difficult to observe a correlation. Although, as will be noted below, within the two major chemotypes identified (AN and DS), there are significantly stronger correlations [*R* ^2^ = 0.76 (AN) and 0.88 (DS), Fig. 7B and C]. Another possible reason for the weak overall correlation is that the various compounds may have different molecular targets in *Shigella* and inhibition of those targets may have differential effectiveness in triggering macrophage pyroptosis and escape versus Caco2 invasion and cell-to-cell spread. Of course, these two assays are conducted using THP-1 macrophages versus Caco2 cells and that may also impact the correlation.

The AN chemotype is clearly the most populated and attractive for follow-up (Fig. 6A). Nitrile groups are widely used in drug discovery and are found in over 50 diverse small molecule drugs (43). The antidepressant selective serotonin reuptake inhibitor(SSRI), escitalopram, the HIV reverse transcriptase inhibitor rilpivirine, and the anticancer drug letrozole (43, 44) are specific examples of AN with very different therapeutic uses and targets. Among our 44 confirmed hit compounds, 7 of the top 10 scoring compounds belong to the AN chemotype, with 3 of these sharing the AN1 scaffold (Table 3). Within the AN1 scaffold, these three compounds have the highest structural similarity. Initial attempts to correlate changes in the structures of the specific compounds within the AN chemotype with their activities failed to reveal any obvious structure-activity relationships. This is likely due to the small numbers of members of the chemotype and the low variance of the activities (as noted previously). However, there is a range of 0.0039–1.08 µM (average = 0.49 µM, SD = 0.33) for the *Shigella* intra-macrophage survival IC_50_s, indicating that the activities do change with changes in structure. While the compounds in the AN chemotype share the AN functionality, their specific scaffold structures vary (Fig. 6A).

The second major chemotype identified is the DS. While bacteriostatic sulfonamides reversibly block the synthesis of folic acid, the sulfonamide functional group is a ubiquitous chemical building block and is found in a very wide variety of marketed drugs (45). The DS chemotype contains nine members in two scaffold sets of three and two members (Fig. 6B). While the DS compounds did not rank quite as highly as the AN compounds, 1 of the top 10 scored compounds is in the DS chemotype (Table 3). The DS compounds range from 0.41 to 1.2 µM (average = 0.76 µM, SD = 0.32) for the *Shigella* intra-macrophage survival IC_50_s. As with the AN chemotype, it is difficult to identify any specific structure-activity relationships. There is more similarity across the DS chemotype than for the AN chemotype; however, there are some distinct DS structures (Fig. 6B).

The PT chemotype consists of the smallest number of compounds (3, Fig. 6C). These compounds are very similar in structure and have *Shigella* intra-macrophage survival IC_50_s averaging 0.40 µM (SD = 0.12). Additionally, 14 individual, “singleton” compounds with an average IC_50_ of 0.68 µM (SD = 0.32) were also identified in our screening and validation process (Fig. 6D). These represent a diversity of chemical structures and many opportunities for potential further study and development.

**Fig 8:**
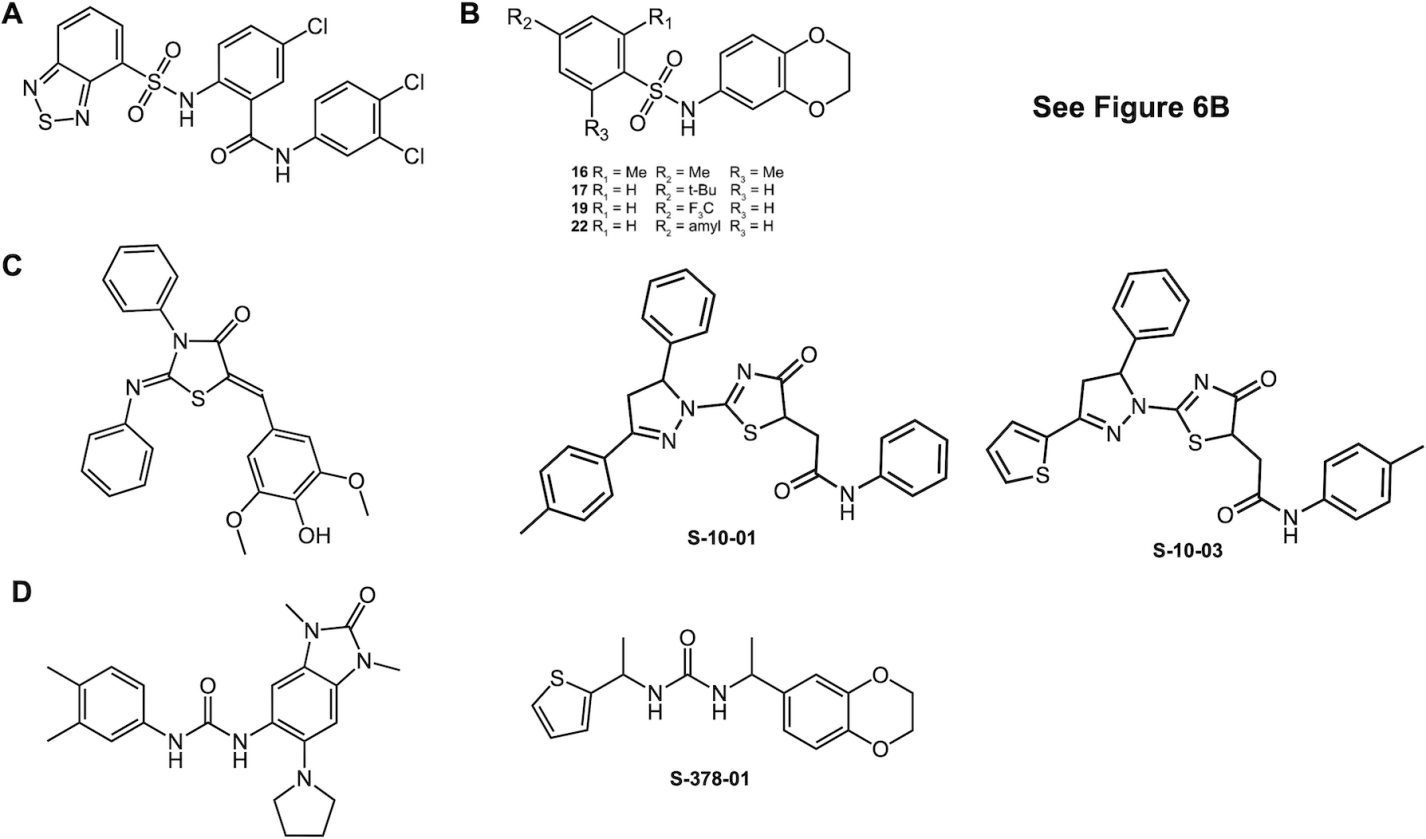
Published type III secretion inhibitors similar to hits reported here. (**A**) DS (50); (**B**) DS (51); (**C**) 2-imino-5-arylidene thiazolidinones (52); (**D**) diarylureas (53).

Targeting virulence is an increasingly attractive and innovative approach to novel antibacterials (46 – 49). Various steps involved in the infection process could be targeted, including adherence, colonization, invasion, quorum sensing, and host defense evasion. Of particular interest are the bacterial targets that are conserved across multiple pathogenic species. One example is the T3SS, which is highly conserved in structure and function among different Gram-negative bacteria. In a screen of ~9,400 compounds, Kauppi et al. found several compounds that inhibit T3SS in *Yersinia* by blocking the yopE promoter (50). One of Kauppi et al.’s hits is a DS (Fig. 8A) similar to our DS chemotype (Fig. 6B). Interestingly, Kim et al. also found a series of DS (Fig. 8B) that inhibit the exoU cytotoxin from *Pseudomonas aeruginosa* (51). A compound (Fig. 8C) similar to S-10-01 and S-10-02 (Fig. 6D) was identified as a T3SS inhibitor in *Salmonella, Yersinia* spp., *P. aeruginosa*, and *Francisella novicida* (52). A compound (Fig. 8D) similar to S-378-01 (Fig. 6D) was reported to block *Yersinia* Yop effector molecules from entering mammalian cells (53). A cyclic diarylheptanoid compound, myricanol, was described as an effective inhibitor of the *Salmonella enterica* T3SS by interfering with the DNA-binding activity of HilD (an AraC-type dominant regulator of virulence) (54). Compounds that inhibit virulence of *M. tuberculosis* were discovered in an HTS of ~28 K compounds against *Mycobacterium marinum* (55). An inhibitor of *Vibrio cholerae* ToxT (the transcription factor that directly regulates expression of two critical virulence factors), named virstatin, was identified from an HTS of 50,000 small molecules (56). To identify an inhibitor of VirF from *S. flexneri*, an HTS of over 140,000 small molecules using VirF-driven/β-galactosidase reporter assay yielded five lead compounds (57, 58). The authors subsequently provided proof of principle that three of five small molecules can attenuate virulence, while the mechanism by which compounds inhibit the VirF transcriptional activation process remained unclear (57). Egan and co-workers also reported a small molecule inhibitor of the *Shigella* VirF, although this did not involve an HTS (59). Using a structure-based approach, a set of novel compounds was designed that inhibit the *V. cholerae* virulence cascade to a greater degree than any other known inhibitor which can potentially serve as novel and effective therapeutics against cholera (60). In addition to the approaches described above, recent studies reported three specific microRNAs as strong inhibitors of *Shigella* infection, specifically bacterial spreading to neighboring cells, while spreading of *Listeria monocytogenes*, which also exploits actin-based motility, remained essentially unaffected by these three microRNAs (61). Our study stands out from those previously reported because few of those studies utilized a phenotypic screen that would identify antivirulence compounds regardless of the specific virulence target, and none were conducted at the extremely large scale, 1.7 M compounds, that we report herein.

Finally, in a review of the GSK database, we find that our 44 hits have no (or less than threshold) activity in the following: *M. tuberculosis, M. bovis, Streptococcus pneumoniae, Staphylococcus aureus, Acinetobacter*, and *Escherichia coli*. The screening for *M. tuberculosis* and *M. bovis* was done against intracellular survival in THP-1 macrophages, similar to our primary HTS assay. The database does show that four of our hits had poor activity against *Leishmania,* two were active against *Plasmodium falciparum,* and one was active against *Trypanosoma cruzi* and *T. brucei*. This suggests that our hits have little or no antibacterial activity and may be selective for *Shigella* or related (e.g., virulence pathways) pathogens; however, further studies are needed.

## MATERIALS AND METHODS

### Bacterial strains and growth conditions

THP-1 (ATCC TIB-202), Caco2 (ATCC HTB-37), HepG2 (ATCC HB-8065), and *Shigella flexneri* serotype 2a strain 2457T (ATCC 700930) were obtained from ATCC. The derivative *S. flexneri* serotype 2a strain 2457T-nl was constructed as described below. The presence of this reporter construct in the derivative strain did not affect bacterial replication in the growth medium and within macrophages. Both strains were routinely grown in Luria-Bertani (LB) broth, aerobically at 37°C. When required, kanamycin was added to the LB medium at final concentration of 20 µg/mL. For the antibacterial assays, Müeller Hinton (MH) medium was used to grow *S. flexneri* 2457T. Experimental work with live *S. flexneri* 2457T (wild-type and derivative strain) was carried out following standard operating procedures in compliance with biosafety level 2 regulations.

#### *S. flexneri* serotype 2a strain 2457T-nl

The synthetic gene Tac + Nanoluc (593 bp) was assembled from synthetic oligonucleotides, and the fragment was inserted into the pMK-RQ plasmid, yielding the 18ADIM2C_Tac + Nanoluc_pMK-RQ (KanR) construct (hereafter referred to as pMK-RQ-nl). *Escherichia coli* K12 DH10B T1R was used for propagation of the plasmid. The plasmid DNA was purified from transformed colonies and sequenced (Dynamimed, Madrid, Spain) for verification. This process was conducted by contract to Invitrogen (Thermo Fisher Scientific, Waltham, MA, USA). Electroporation of pMK-RQ-nl into *S. flexneri* 2457T was performed according to a standard protocol (62) yielding the *S. flexneri* serotype 2a strain 2457T-nl used in this study.

#### Eukaryotic cell lines and growth conditions

The published intracellular HTS for finding new antituberculosis compounds active in human macrophages (35) was used as a guide for the THP-1 cell growth, differentiation, and density determination. The human monocytic leukemia cell line THP-1 (ATCC TIB-202) was maintained and expanded in the RPMI 1640 medium (Sigma-Aldrich, St. Louis, MO, USA) supplemented with 10% heat-inactivated fetal bovine serum (FBS) (Gibco, Waltham, MA, USA), 2 mM L-glutamine (Sigma-Aldrich, St. Louis, MO, USA), and 1 mM sodium pyruvate (Sigma-Aldrich) at 37°C in a humidified atmosphere with 5% CO_2_ with a slow roller bottle rotation. The human epithelial colorectal adenocarcinoma Caco2 (ATCC HTB-37) and hepatocyte carcinoma HepG2 (ATCC HB-8065) cell lines were grown in the Minimum Essential Medium (MEM) supplemented with 10% heat-inactivated FBS (Gibco) and 2 mM L-glutamine (Sigma-Aldrich) at 37°C in a humidified atmosphere with 5% CO_2_. When required, 100 U/mL penicillin/100 µg/mL streptomycin solution (Sigma-Aldrich) was added to the tissue cultures to protect them from microbial contamination only during cell growth and were removed prior to *Shigella* infections studies. Cell lines used in this study (THP-1, Caco2, and HepG2) were treated according to GSK policies for management of human biological samples.

#### Compound dispensation for HTS

For the assay validation, pilot HTS (see below) and primary HTS, compounds from GSKʼs ~1.7 M compound screening collection were robotically dispensed in 1,536-well polystyrene plates (Black, HiBase, μClear, Greiner Bio-One, Monore, NC, USA) at a final concentration of 10 µM. In general, 1,408 compounds were dispensed per plate, arranged in 44 columns with the remaining 4 columns used for plate controls (columns 11 and 12: 0.5% DMSO as negative control and columns 35 and 36: 20 µM moxifloxacin as positive control). Following the HTS screen, confirmation assays were conducted using the same plate format and compound dispensing as described above. For dose-response and cytotoxicity assays, compounds were robotically dispensed in 384-well polystyrene plates at 11 concentrations using threefold serial dilutions starting at 200 or 100 µM (depending on the screening requirements; details below). The serial dilutions were dispensed along the row with the appropriate plate controls (column 6: DMSO as negative control and column 18: moxifloxacin [20 µM, for dose-response assay] or doxorubicin [10 µM, for cytotoxicity assays] as positive control). Compounds were dispensed by Sample Management (Sample Management, Stevenage, UK, and Sample Management, Upper Providence, RI, USA) and stored at −20°C until needed.

### *S. flexneri* 2457T-nl intracellular survival in THP-1 macrophage (*Shigella* intra-macrophage survival) assay development

#### Assay protocol

The experimental protocol for the *Shigella* intra-macrophage survival screen is divided into three time segments: (i) THP-1 macrophage and bacteria preparation 1 day prior to compound screen assay, (ii) THP-1 differentiation, invasion, gentamicin treatment, dispensing of infected cells onto plates pre-dispensed with compounds to be tested, and the beginning of their incubation performed on the same day, and (iii) dispensing of the Nano-Luc substrate and luminescence readout after 18 hours of incubation. The following steps in the *Shigella* intra-macrophage survival screen protocol, the time required to perform one run of assay, and the method efficiency are shown in Fig. S1.

##### Time segment 1

One day prior to screening, THP-1 cells were filtered (using 40 µm filters) to remove cell aggregates (clusters) and were seeded (~8 × 10^5^ cells/mL) into a roller bottle (CELLMASTER Cell Culture Roller Bottles, Greiner Bio-One) at 37°C in a humidified atmosphere with 5% CO_2_ and slow rotation. Also, an overnight culture of *S. flexneri* 2457T-nl was inoculated with a single colony and grown in LB medium supplemented with 20 µg/mL kanamycin at 37°C with aeration.

##### Time segment 2

The monocytic differentiation of the THP-1 cells was induced with PMA (Sigma-Aldrich) at the final concentration 40 ng/mL for 4 hours at 37°C in a humidified atmosphere with 5% CO_2_ and slow rotation. Following differentiation, cells were pelleted by centrifugation [1,300 rpm, 5 minutes, and room temperature (RT)] and resuspended with equal volume of fresh pre-warmed supplemented RPMI 1640 medium to remove any trace of PMA, and macrophages were used immediately for bacterial invasion. The bacterial overnight culture of *S. flexneri* 2457T-nl was diluted 100-fold in LB medium without antibiotic (3 hours prior to infection) and grown to mid-log phase at 37°C with aeration to an optical density (OD_600_) between 0.5 and 0.6. Bacterial cells were then harvested by centrifugation (4,500 rpm, 10 minutes, and RT) and suspended in the same volume of pre-warmed supplemented RPMI 1640 medium. The bacteria, at the desired MOI, were added to the roller bottles containing the prepared THP-1 macrophages and incubated for 2 hours at 37°C in a humidified atmosphere with 5% CO_2_ and slow rotation. After bacterial invasion into macrophages, the suspension was washed by centrifugation (1,300 rpm, 5 minutes, and RT) and suspended in equal volume of pre-warmed supplemented RPMI 1640 medium containing 100 µg/mL gentamicin for 30 minutes at 37°C in a humidified atmosphere with 5% CO_2_ and slow rotation. This suspension was subsequently washed twice with pre-warmed supplemented RPMI 1640 medium (first wash in the equal volume and second wash in half of volume) and filtered (using 40 µm filters) to remove cell aggregates. The macrophages were counted using hemocytometer (Neubauer chamber) and suspended in the appropriate volume of pre-warmed supplemented RPMI 1640 medium, resulting in 5–7 × 10^5^ cell/mL. The assay was adapted to the 1,536-well plate format for the HTS. A multidrop dispenser with the small tube metal tip cassette (Thermo Fisher Scientific) was used to seed 5 µL of the cell suspension to plates pre-dispensed with compounds to be tested that were transferred to RT 1–1.5 hours before adding infected cells. Plates were incubated for 18 hours at 37°C in a humidified atmosphere with 5% CO_2_.

##### Time segment 3

After incubation, the bacterial count is quantified by luminescence readout. Luminescence correlated linearly with the number of viable bacteria. The Nano-Luc substrate solution was prepared according to the manufacturer (Nano-Glo Luciferase Assay System, Promega, Madison, WI, USA). A multidrop dispenser with the small tube metal tip cassette (Thermo Fisher Scientific) was used to dispense 2 µL of substrate per well, and plates were stored in the dark prior to reading. Luminescence was read (0.1 s/well) on a microplate reader (EnVision, PerkinElmer) after 30 minutes of incubation at RT.

### MOI optimization

In order to optimize the ratio of the number of bacteria and macrophages, different MOIs were tested. The bacteria in RPMI 1640 medium at MOIs 50, 20, 8, and 1 were incubated with differentiated THP-1 cells for 2 hours (see paragraph below). The cell suspensions were washed two times and incubated in fresh pre-warmed RPMI 1640 medium supplemented with 100 µg/mL gentamicin for 30 minutes at 37°C in a humidified atmosphere with 5% CO_2_ with a slow roller bottle rotation to kill the extracellular bacteria. Following this incubation time, cells were washed twice and lysed in 1% Triton X-100 for 3 minutes at RT. Bacteria were collected and suspended in phosphate-buffered saline (PBS, Sigma-Aldrich) for quantification of the number of intracellular bacteria by CFU on agar plates. Experiments were carried out in duplicate.

### Initial optimization of infection time

Using the previously established MOI of 8, different infection times were tested (0.5, 1, 1.5, 2, 4, and 24 hours) according to the above protocol. The number of intracellular bacteria was determined by CFU on agar plates. Experiments were carried out in duplicate.

### Gentamicin treatment

Infected macrophages are treated with gentamicin to eliminate any extracellular bacteria that would confound the assay. The importance of gentamicin treatment was tested to ensure that no bacteria remain outside the cells after THP-1 macrophage invasion and to determine that exposure of infected macrophages to gentamicin at concentration 100 µg/mL for 30 minutes does not affect the number of intracellular bacteria. Bacteria in RPMI 1640 medium at MOI 8 were incubated with differentiated THP-1 cells for 2 hours. The same protocol described above was performed, including a second set of infected macrophages without gentamicin treatment. Both gentamicin-treated and gentamicin-untreated cell suspensions were washed twice and re-suspended in PBS to obtain the number of extracellular bacteria by CFU on agar plates. Using the same samples, cells were lysed in 1% Triton X-100 for 3 minutes at RT, and the number of intracellular bacteria was quantified by CFU on agar plates. Experiment was carried out in duplicate.

### Infection incubation time optimization for HTS

The differentiated THP-1 macrophages were separated into two roller bottles: in the first, THP-1 macrophages were infected with *S. flexneri* 2457T-nl, while cells in the second was used as non-infected macrophages (control). This experiment was performed under the same conditions as described above. After the gentamicin treatment, cell suspensions were subsequently washed twice with pre-warmed supplemented RPMI 1640 medium and filtered through 40 µm filters. The macrophages were counted and suspended in the appropriate volumes of pre-warmed supplemented RPMI 1640 medium, resulting in 5–7 × 10^5^ cell/mL.

The aliquots in duplicate (100 µL) were taken from both groups (infected and non-infected THP-1 macrophages) into the seven separated 96-well plates. Plates were incubated for 3, 4, 16, 18, 24, 40, and 48 hours at 37°C in a humidified atmosphere with 5% CO_2_. In each of the listed time points, the supernatants were removed from the samples, and wells were washed twice with PBS (using 100 and 150 µL). That plate was transferred and stored at −70°C until usage and process were repeated for all time points. A Caspase-1 assay was performed according to manufacturer’s instructions (Caspase 1 Assay Kit, Fluorometric, Abcam, Cambridge, UK).

The aliquots in duplicate (100 µL) were also taken from both groups (infected and non-infected THP-1 macrophages) and dispensed onto microplates (Cellcarrier 96 Ultra Microplates, PerkinElmer). The plate was incubated at 3, 4, 18, and 36 hours, and, in each time point, the supernatants were removed from the samples, and wells were washed twice with PBS (using 100 and 150 µL). Infected and non-infected macrophages were fixed with a formaldehyde solution (final concentration 8% vol/vol) and subsequent incubation at RT for 10 minutes. Then, formaldehyde solution was removed, and cells were washed twice with 100 µL of PBS. Nuclei of the THP-1 macrophages were stained with 5 µM DRAQ5 far-red fluorescent DNA dye (Biostatus) with 647 nm excitation wavelengths in dark. Single plane images were acquired on an Opera Phenix high-content screening system (PerkinElmer) using a 20× water immersion objective in confocal mode. Three fields per well were acquired for reliable statistical analysis, and images were analyzed using Harmony (PerkinElmer) HC imaging software.

### *Shigella* intra-macrophage survival assay validation

#### Reproducibility

To determine the reproducibility of the optimized HTS assay, 5 µL of infected THP-1 macrophages was dispensed into 1,536-well plates pre-dispensed with DMSO at a final concentration 0.5% and moxifloxacin at a final concentration 20 µM. Plates are incubated for 18 hours at 37°C in a humidified atmosphere with 5% CO_2_, and luciferase readout was done as described above. This experiment was repeated 10 independent times.

#### Tissue-permeable antibiotic controls

To examine the performance of HTS, we tested the commercially available drugs with known activity against Gram-negative bacteria. This set of compounds represents a wide variety of chemotypes: quinolone (ciprofloxacin), β-lactam (mecillinam and pivmecillinam), macrolide (azithromycin), cephalosporin (ceftriaxone), and LpxC inhibitor (PF-5081090).

#### Pilot screening

Prior to initiating the HTS, a validation set of ~10 K compounds (selected from the 1.7 M compound screening collection) was used for HTS methodology validation. Additionally, a pilot screen of ~58 K compounds from the 1.7 M compound collection was performed and progressed through dose response to establish the ability of the assay to identify hit compounds prior to committing to the 1.7 M HTS.

### HTS progression cascade

#### HTS of 1.7 M compounds in the *Shigella* intra-macrophage survival assay

This screen was performed according to the intra-macrophage survival assay protocol described above. A total of 1,220 plates were assayed in 53 HTS rounds (the total number of plates that contained the entire compound library was 1,428; 208 of them were not taken for further analysis due to technical failures and lack of set criteria).

#### Confirmation assay

The confirmation screen was performed in duplicate using a new set of samples as described above.

#### *Shigella* intra-macrophage survival dose-response assay

The dose-response assay was performed following the above protocol using different plate formats. A multidrop dispenser with the standard tube dispensing cassette (Thermo Fisher Scientific) was used to seed 25 µL cell suspension to 384-well plates pre-dispensed with compounds at 11 concentrations using threefold serial dilution with a maximum concentration of 200 mΜ. After 18 hours of incubation, 25 µL of Nano-Luc substrate per well was dispensed, and plates were stored in a dark place prior to reading. Luminescence was read (0.1 s/well) on a microplate reader (EnVision, PerkinElmer) after 30 minutes incubation time at RT. The experiment was done in duplicate.

#### *Shigella* antibacterial assay

An antibacterial assay against *S. flexneri* 2457T was performed according to the previously described protocol (40). The antibacterial assay was used to determine the MIC_90_ values of potential inhibitors for *S. flexneri* 2457T. The assay measures the effect of compounds on the bacteria using resazurin (Thermo Fisher Scientific) measurement as surrogate of bacterial viability. Briefly, to perform the extracellular dose responses, compounds were pre-dispensed in DMSO, and threefold serial dilutions were made for each compound into 384-well plate format. Bacterial inoculum for the assay was prepared in MH medium, resulting at 1 × 10^6^ CFU/mL, and 5 µL of this culture was dispensed per well. Then, plates were incubated at 37°C overnight. The resazurin reagent (10 µL) was added to each well followed by an incubation at RT and darkness for 3 hours. The fluorescence intensity of the assay plates was measured using microplate reader (EnVision, PerkinElmer). For MIC_90_ determination, data were normalized by using DMSO at a final concentration of 0.5% as the negative control (100% bacterial growth) and moxifloxacin at a final concentration of 20 µM as the positive control (0% bacterial growth). The experiment was done in duplicate.

### Hit compound filtering

One of the most important aspects of a successful screening campaign is the post-HTS data processing and hit compound selection (63). The compound selection in this study was based on set of parameters obtained in the actual experimental screen (experimental parameters) and cheminformatics and knowledge-based analyses (calculated parameters). The values for inhibition frequency index (IFI), property forecast index (PFI), FaSSIF, and hERG channel inhibition (hERG) were obtained from the GSK proprietary database. In addition, chemical structural novelty was an important criterion for hit compound filtering. Hit compounds that either are known antibiotics or are analogs of known antibiotics were excluded in the filtering.

#### Experimental parameters

The first step in the compound prioritization was based on the potency with the thresholds of % inhibition as shown in Fig. 2. In the next step, the chemical/structural efficiencies of the active compounds were estimated. The ligand efficiency (LE) is a parameter used in drug design which normalizes a compound’s efficiency on a per-heavy-atom basis [LE = 1.37 × pIC_50_ / # of heavy atoms; (64)]. The lipophilic LE (LLE) is another parameter used in drug design which attenuates a compound’s potency based upon its lipophilicity [LLE = pIC_50_ − cLogD_7.4_; (65)]. To exclude promiscuous and non-specific compounds from the analysis, IFI was calculated. This is the relative frequency with which a compound has scored more than 50% inhibition in an HTS assay, calculated according to reference (66).

#### Aqueous solubility

The PFI was used to estimate compound solubility in aqueous solution at pH 7.4: PFI = Chrom Log D_7.4_ + # aromatic rings (67). The chromatographic LogD at pH 7.4 (Chrom LogD_7.4_) is calculated from the experimentally determinchromatographic hydrophobicity index (CHI) as follows: Chrom Log D_pH_ = (0.0857) x CHI_pH_ − 2.0). PFI does not consider any solution components other than water at pH 7.4.

#### FaSSIF solubility classification (68)

Experimentally determined solubility data in fasted state simulated intestinal fluid [FaSSIF-V3 (69)] were binned into the following classifications: VH—very high, H—high, MH—moderately high, L—low, ML—moderately low, VL—very low. For scoring purposes (see below), these bins were assigned numeric values as follows: VH = 3, H = 2, MH = 1, L = −1, ML = −2, and VL = −3.

#### hERG channel inhibition

Lipophilicity plays a dominant role in promoting binding to unwanted drug targets (70). Lipophilic bases can cause cardiovascular toxicological effects by binding to the hERG (human ether-ago-go-related potassium channel protein; also known as KCNH2) ion channel (71) and tissue toxicity by promoting cellular phospholipidosis. hERG inhibition predictions were binned into the following classifications: H—high, M—medium, and L—low. For scoring purposes (see below), these bins were assigned numeric values as follows: H = −3, M = 1, and L = 3.

#### Pan-assay interference

Pan-assay interference (PAINS) compounds were initially a series of compounds identified by Baell et al*.* (72) that showed bioactivity in multiple AlphaScreen HTS campaigns, irrespective of the biological target. Compounds containing the PAINS substructures should generally be considered unsuitable as leads for chemical probes or medicinal chemistry optimization. The chemical mechanism(s) behind their promiscuous bioactivities vary depending on chemotype. The 44 final hits were screened through the SwissADME web tool (http://swissadme.ch/), and only one singleton compound had a PAINS alert, and we elected to retain that compound.

#### Nano-Luc inhibition

The protocol was identical to the *Shigella* intra-macrophage survival dose-response assay discussed above with the following modifications: 50 nL of each compound dilution was dispensed into each well of a 1,536-well plate and 5 µL of a fresh culture of 1 × 10^6^ CFU/mL of the *S. flexneri* 2457T-nl reporter strain as used for the initial HTS in MH medium was added to each well. After 18 hours of incubation at 37°C, 5 µL of the Nano-Glo reagents was added and the plates read as described above.

#### The inhibition of *Shigella* propagation in Caco2 cells assay

The inhibition of *Shigella* propagation in Caco2 cells assay was performed following the previously described protocol based on microcarrier cell culture which models the *in vivo* three-dimensional environment with the minor modifications. This protocol will be described in a forthcoming paper (V. Phat, A. Lim, S. Lozano, C. De Cozar-Gallardo, M. Castellote-Alvaro, J. Rodrigues, A. Villegas, D. Alvarez, E. Alvaro, L. Ballell-Pages, S. Baker, unpublished data) and is available upon request (see Data Availability Statement).

### Cytotoxicity assays

These assays have been performed to measure influence of compounds administered to human THP-1 monocytes differentiated to macrophages, human epithelial colorectal adenocarcinoma Caco2, and hepatocyte carcinoma HepG2 cell lines. The effect of the compounds on cell viability was analyzed using the resazurin measurement as surrogate parameter for overall cellular viability.

#### Cytotoxic influence of compounds on THP-1 macrophages

The assay was designed based on previously described protocols with slight modifications (73, 74). Briefly, a suspension of THP-1 with 5 × 10^5^ cells/mL was prepared using the complete RPMI-1640 medium and PMA was added to the cells in a final concentration of 40 ng/mL. Cells were incubated at 37°C in a humidified atmosphere with 5% CO_2_ and left to differentiate for 4 hours. Then, cells were washed with complete RPMI-1640 medium, centrifuged (1,500 rpm, 5 minutes, and RT), filtrated, and adjusted cell density to 2 × 10^5^ cells/mL. An amount of 50 µL of this suspension (10,000 cells/well) of differentiated cells was transferred into Greiner black 384-well plates using a Multidrop Combi dispenser. Those plates already contain compounds performing threefold serial dilution with a top concentration of 100 µM. Cells are left at 37°C, with 5% CO_2_ in a humidified incubator in the presence of compound for 18–20 hours. Then, 25 µL resazurin reagent was added to each well using a Multidrop Combi dispenser, and plates were shaken followed by an incubation under the above-mentioned conditions and darkness for 4 hours. The fluorescence intensity of the assay plates was measured using a microplate reader (EnVision, PerkinElmer) at a wavelength of 590 nm (emission) and 530 nm (excitation). The effect of a given compound is estimated as: % cytotoxicity = 100 × [(min cytotoxicity control − data)/(min cytotoxicity control − max cytotoxicity control)]. Complete cytotoxicity of cell culture is achieved in max cytotoxicity control in the presence of 10 µM doxorubicin, while min cytotoxicity control provides the measure of maximum cell health, achieved in cell culture media supplemented with 0.5% DMSO. The cytotoxicity results were expressed as Tox_50_ values (IC_50_). The experiment was done in duplicate.

#### Cytotoxic influence of compounds on HepG2 and Caco2 cells

This assay was a slight modification of that described above for THP-1. Briefly, independent cultures of actively growing HepG2 and Caco2 cells were removed from a T-175 TC flask, harvested by centrifugation (1,500 rpm, 5 min, RT), suspended in the Eagle’s MEM (containing 10% FBS), filtered (using 40 µm filters), and counted using a cell counter. Cell suspensions of 25 µL, at a final density of 2 × 10^5^ cells/mL, were dispensed using a Multidrop Combi dispenser into Greiner black 384-well plates containing 10 serial threefold dilutions (starting at 100 µM) of test compounds. Plates were stored at 37°C with 5% CO_2_ in a humidified incubator for 18–20 hours. Resazurin reagent (25 µL) was then added to each well followed by an incubation under the above conditions and darkness for 4 hours. The fluorescence intensities were measured, and the data were processed to determine the compounds’ toxicities following the protocol described above. The experiment was done in duplicate.

### Data analysis

Data from the HTS, confirmation screening, dose response, and cytotoxicity experiments were analyzed with the IDBS Activity—Base XE (ID Business Solution Limited, Woking, UK). The quality of the HTS was assessed by determining the Z′ parameter (75) as follows:


$$Z^{\prime }=1-3\left(\frac{\sigma^{+}\;+\;\sigma^{-}}{\mu^{+}\;-\;\mu^{-}}\right)$$

where *µ^+^* and *µ* ^−^ are the means of the positive and negative controls, and *σ^+^* and *σ* ^−^ are the standard deviations of the positive and negative controls, respectively. A Z′ factor greater than 0.4 indicates that the assay is suitable for HTS and the data are reliable.

In the HTS and confirmation assays, the percent of inhibition for each compound was calculated relative to the assay plate controls using the formula:


$$\%\;inhibition=\left(\frac{X\;-\;control^{-}}{control^{+}\;-\;control^{-}}\right)\times 100$$

where *X* is the sample data value, *control^–^* is the average of DMSO signal, and *control^+^* is the average of moxifloxacin signal.

For inhibitory concentration (IC_50_ and MIC_90_) determination, dose-response curves were fitted using IDBS Activity Base and displayed using Spotfire (TIBCO Spotfire). The IC_50_ was determined as follows, where the IC_50_ is in molar units.


$$IC_{50}=antilog_{10}(-pIC_{50})$$

#### Comparative analyses of HTS results for *Shigella* intra-macrophage survival and *Shigella* antibacterial 1.7 M compound screens

The plots of comparative data analyses of *Shigella* (2457T-nl) intra-macrophage survival HTS and *Shigella* (2457T) antibacterial HTS were created using “ggplot2” (https://ggplot2.tidyverse.org/; which is based on statistical programming language R). In the data analysis process, it was determined that 1.65 M unique compounds were screened in the *Shigella* intra-macrophage survival HTS and 1.73 M compounds in the *Shigella* antibacterial HTS. A total of 1.43 M compounds were screened in both HTS.

### Hit compound priority scoring system

A priority scoring system was developed to rank the hit compounds without consideration of their chemical structures. This scoring system considers: (i) an activity score based on potency in the *Shigella* intra-macrophage survival assay, (ii) physical parameter score based on molar mass, solubility, LE, and LLE, and (iii) “off-target” score based on cytotoxicity, hERG channel inhibition, and promiscuity. The weighting values for each parameter, determined by multiplying a “metric normalization factor” by a “priority weighting factor,” are presented in Table 2.

## Data Availability

The methodology for the *Shigella* propagation in Caco2 cells assay will be described in a forthcoming paper (Phat et al., unpublished). However, the methodology is available upon request by emailing to Tres Cantos Open Lab Foundation (info@openlabfoundation.org).

## SUPPLEMENTAL MATERIAL

Supplemental information msphere.00154-23-s0001.pdf Table S1 and Fig. S1 to S4.

ASM does not own the copyrights to Supplemental Material that may be linked to, or accessed through, an article. The authors have granted ASM a non-exclusive, world-wide license to publish the Supplemental Material files. Please contact the corresponding author directly for reuse.
