# Supplementary material for: Novel inhibitors that target bacterial virulence identified via HTS against intra-macrophage survival of Shigella flexneri
Source: mSphere. 2023 Aug 11;8(5):e00154-23. doi: 10.1128/msphere.00154-23 (PMC10597453; doi:10.1128/msphere.00154-23)
Supplement: Supplemental information — Table S1 and Fig. S1 to S4. [file msphere.00154-23-s0001.pdf]

Supplementary Information

Novel inhibitors that target bacterial virulence identified via HTS against intra-macrophage survival of *Shigella flexneri*

Marija Miljkovic[1,2,#], Sonia Lozano[2,#,*], Isabel Castellote[2], Cristina de Cózar[2], Ana I. Villegas-Moreno[2], Pablo Gamallo[2], Dolores Jimenez-Alfaro Martinez[2], Elena Fernández-Álvaro[2], Lluis Ballell[2], George A. Garcia[1,*]

[1] Department of Medical Chemistry, College of Pharmacy, University of Michigan, Ann Arbor, Michigan, United States of America

[2] Global Health Discovery Incubator, Diseases of the Developing World (DDW), GlaxoSmithKline, Tres Cantos, Madrid 28760, Spain

[#]Authors with equal contribution

*Correspondence to: gagarcia@umich.edu or sonia.a.lozano@gsk.com

**Antibiotic Class & Name**	***Shigella* Intra-macrophage Survival IC_{50} (µM)**	***Shigella* Antibacterial MIC_{90} (µM)**
**Quinolone**		
Ciprofloxacin	0.007	0.07
**β-Lactam**		
Mecillinam	0.7	0.2
Pivmecillinam	0.9	1
**Macrolide**		
Azithromycin	0.08	10
**Cephalosporin**		
Ceftriaxone	0.01	0.1
**LpxC inhibitor**		
PF-5081090 (Pfizer 2012)	0.06	0.2

Supplementary Table 1. *In vitro* activities of commercially available antibiotics against *S. flexneri* 2457T obtained in intra-macrophage survival (luciferase assay) and antibacterial (resazurin assay) assays.

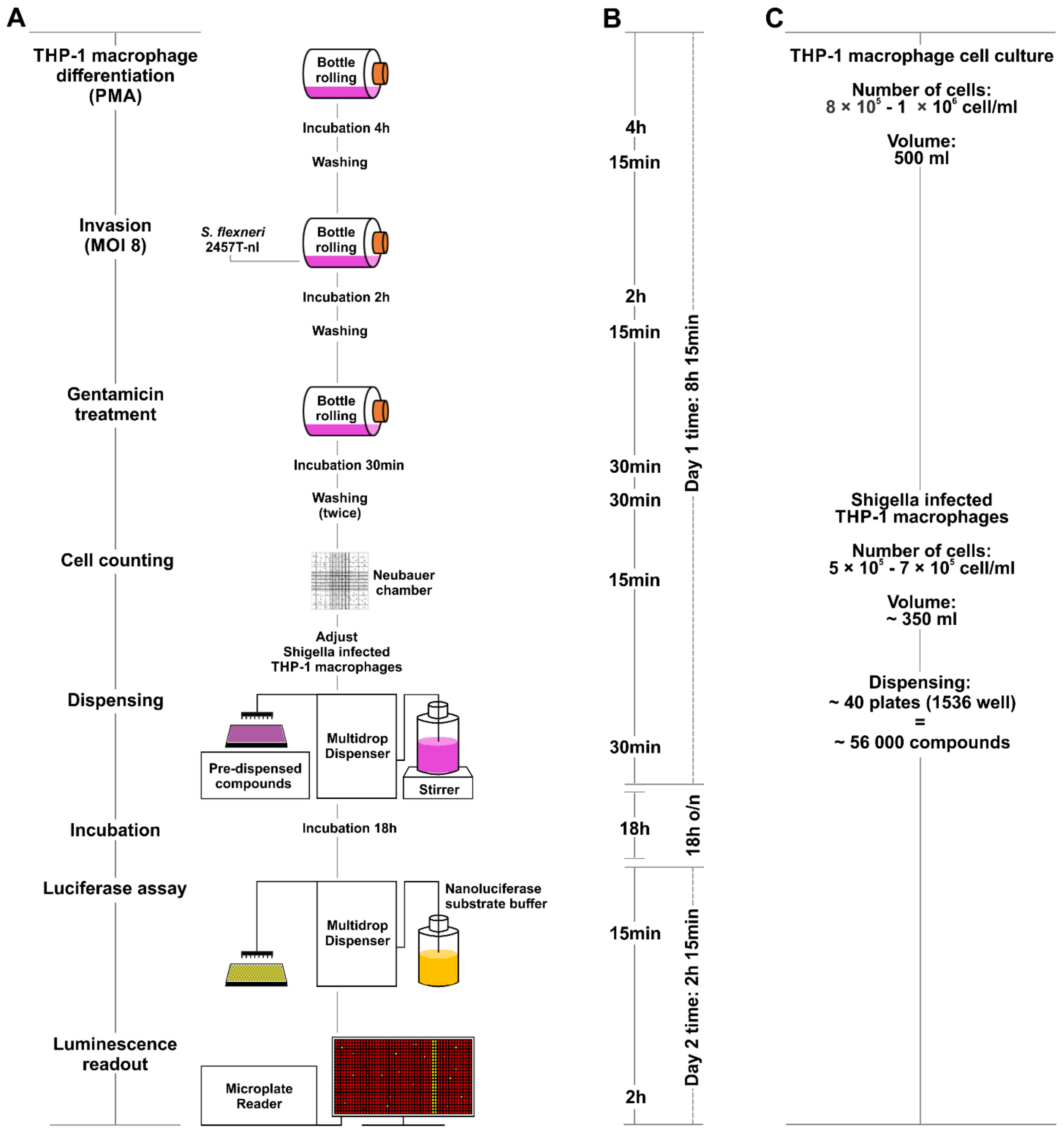

Supplementary Figure 1. ***Shigella* intracellular survival in THP-1 macrophage HTS protocol**.

A) Schematic representation of experimental protocol. B) Time required to perform one round of assay. C) Method efficiency.

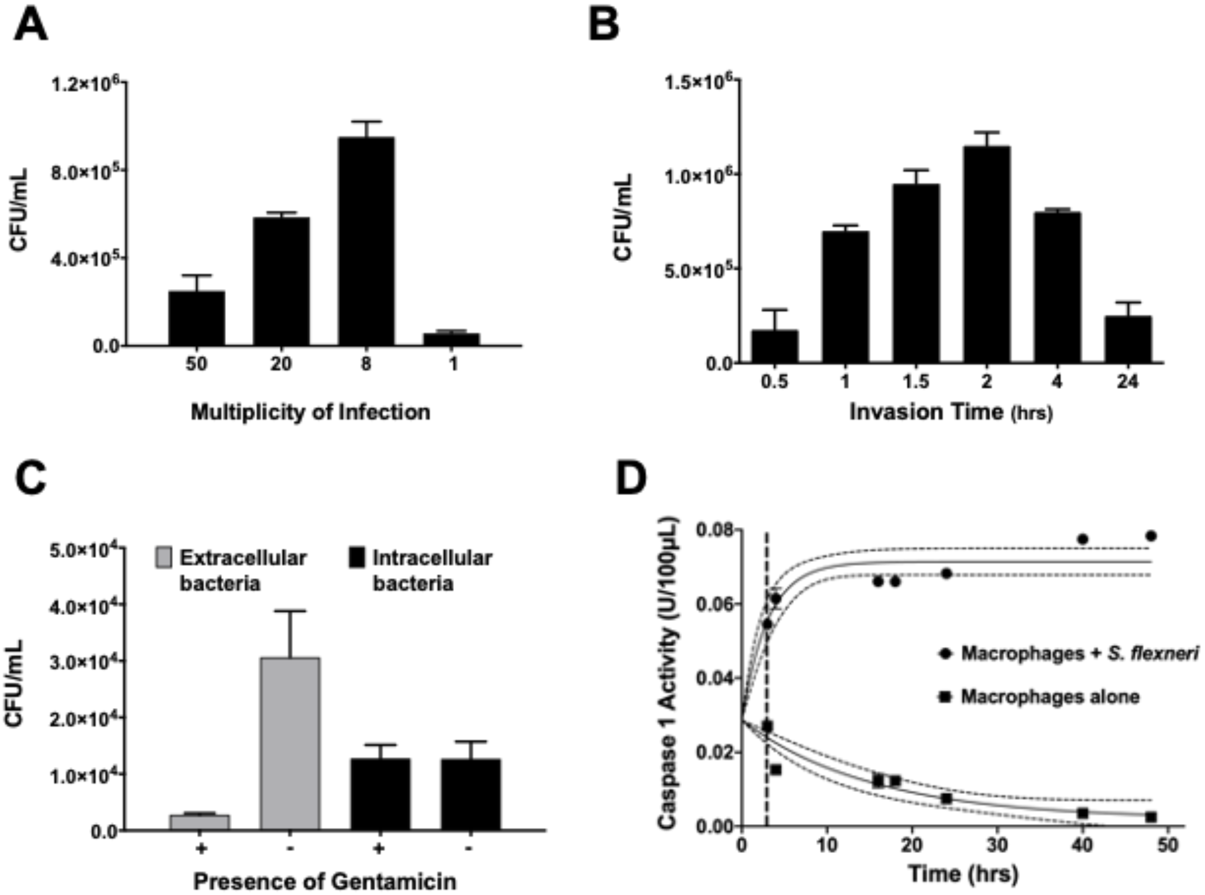

Supplementary Figure 2. ***Shigella* intracellular survival in THP-1 macrophage HTS methodology development.**

A) Optimization of the multiplicity of infection. B) Initial optimization of infection time based on CFU at MOI = 8. C) Importance of gentamicin (100 µg/mL) treatment. D) Determination of optimal incubation time (*S. flexneri* 2457T-nl infected THP-1 macrophages and compounds, round symbols) based on measurements of Caspase-1 activity. The first time point that could be taken was at 3 h. The “macrophages alone” data (square symbols) were fit and the extrapolated zero time intercept was used as a fixed intercept in the “macrophages + *S. flexneri*“ fit. The data were fit via nonlinear regression to a one-phase exponential association model: Y = Y0 + (Plateau-Y0)*(1-exp(-k*x)). The standard deviations of the fit are plotted in dashed lines.

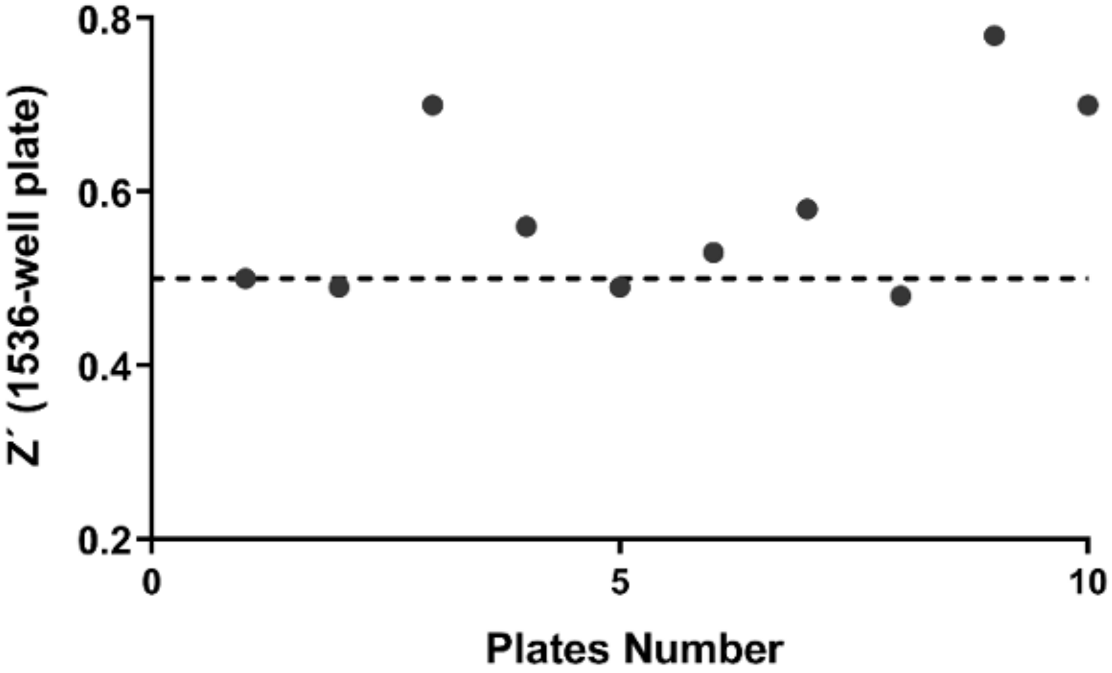

Supplementary Figure 3. ***Shigella* intracellular survival in THP-1 macrophage HTS format assay validation.**

Quality of the 10 independent runs of assay using 1536-well plate was determined by Z' parameter. Plates contain only negative control (DMSO; 0 % inhibition of *S. flexneri* survival) and positive control (moxifloxacin; 100 % inhibition of *S. flexneri* survival). The equation for Z' parameter is described in Methods.

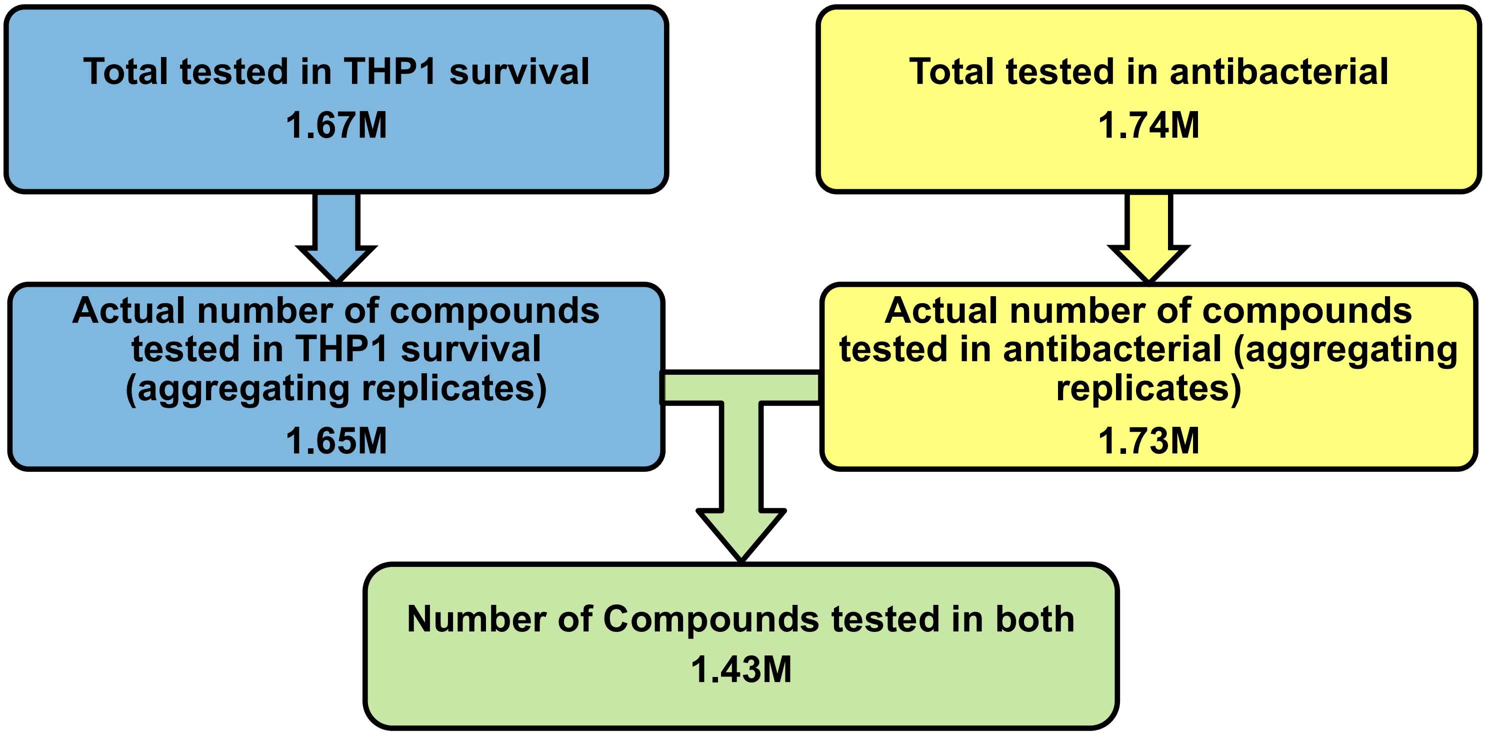

Supplementary Figure 4. Schematic representation of numbers of compounds tested in intracellular survival in THP-1 macrophage HTS and *Shigella* antibacterial HTS including overlap.
